# Heteroatom‐Mediated Covalent Organic Framework Membranes With Nitrogen‐Enriched Nanochannels for Efficient Desalination

**DOI:** 10.1002/advs.77400

**Published:** 2026-09-13

**Authors:** Guishan Hu, Muning Chen, Yunqiu Zhou, Yu Zhen, Miaomiao Tian, Yatao Zhang, Jingwei Hou, Junyong Zhu, Yong Wang

**Affiliations:** ^1^ School of Chemical Engineering Zhengzhou University Zhengzhou Henan China; ^2^ School of Ecology and Environment Zhengzhou University Zhengzhou Henan China; ^3^ School of Chemical Engineering The University of Queensland StLucia Queensland Australia; ^4^ School of Energy and Environment Southeast University Nanjing Jiangsu China

**Keywords:** COF membranes, heteroatom precursor engineering, interfacial polymerization, nitrogen‐enriched nanochannels, water/ion sieving

## Abstract

Covalent organic framework (COF) membranes synthesized via scalable interfacial polymerization (IP) show significant promise for water purification and desalination. However, random monomer diffusion and rapid polymerization kinetics often result in limited crystallinity and disordered pore architectures within COF membranes, compromising their separation efficacy. Here we address this challenge by introducing a heteroatom precursor engineering strategy to develop ultra‐microporous, highly crystalline COF membranes tailored for monovalent ion rejection. The strategic nitrogen substitution within the aromatic ring of hydrazide monomers enables gradient reduction of their oil–water partition coefficients and reactivity, thereby finely reducing the interfacial reaction kinetics. This inhibited diffusion‐reaction behavior facilitates the self‐healing of nascent polymeric chains into ordered networked structures, yielding crystalline nanofilms with controlled ultrafine pore width (0.40–0.68 nm). Augmented by hetero‐nitrogen‐induced electrostatic interaction and hydrogen‐bond recognition, the resulting ultra‐microporous COF (Tp‐PDHz) membranes exhibited exceptional monovalent‐ion removal capability (e.g., *R*_NaCl_ = 97.5%, *R*_LiCl_ = 98.0%), and a high water permeance of 1.2 L m^−2^ h^−1^ bar^−1^. Molecular dynamics simulations reveal that nitrogen‐enriched nanochannels accelerate water transport via reinforced hydrogen‐bond networks while simultaneously increasing the energy barrier for ion permeation. This work provides a molecular‐level roadmap for the rational design of high‐performance COF membranes in next‐generation water desalination and purification.

## Graphical abstract

Heteroatom precursor engineering serves as a molecular‐level strategy to tailor hydrazide monomer reactivity and diffusion kinetics, enabling controlled assembly of highly crystalline COF membranes with ultramicroporous channels. The nitrogen‐enriched nanochannels deliver high water/ion selectivity via simultaneous water transport acceleration and ion barrier elevation.

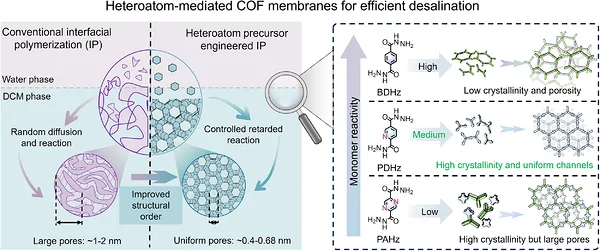

## 1 Introduction

Water scarcity has escalated into a pressing global crisis, posing a direct threat to sustainable development. Despite the ubiquity of water on Earth, the freshwater fraction remains a mere 2.5%, a resource whose stochastic distribution is insufficient to satisfy burgeoning global [1, 2]. Currently, over 40% of the global population experiences water stress, with more than 700 million people lacking access to potable water [3]. This crisis necessitates the transition toward non‐conventional water sources—including seawater, brackish water, and industrial effluent—via advanced water purification technologies [4, 5]. Membrane‐based separations have emerged as the preferred energy‐efficient and sustainable alternatives to thermal processes [6, 7, 8]. While conventional polymer membranes offer promising ion sieving, they are frequently limited by disordered pore architectures and limited tunability, which compromise their separation efficacy [9]. The advent of reticular chemistry enables a paradigm shift toward the bottom‐up assembly of framework‐based membranes. In particular, covalent organic frameworks (COFs) have surfaced as a pivotal material class, offering ordered and interconnected pore architectures that circumvent the ion transport limitations inherent in disordered membrane matrices [10, 11, 12].

Currently, large‐area COF membranes with nanoscale thickness can be synthesized via interfacial polymerization (IP), a long‐standing industrial benchmark for fabricating thin‐film composite membranes [13, 14]. This well‐established process leverages rapid reaction kinetics and inherent self‐limiting characteristics to produce continuous, ultrathin films at confined liquid–liquid or liquid–solid interfaces [15, 16, 17]. This bottom‐up approach precludes the problematic processing of bulk COF powders into functional continuous membranes. Furthermore, the IP technique is amenable to diverse precursor pairs and proceeds under ambient conditions, offering high versatility, cost‐efficiency, and scalability for COF membrane synthesis [18, 19]. Of significant importance is the inherent modularity of the IP platform, which permits the fine‐tuning of pore structure and surface chemical characteristics via rational precursor selection or functionalization [20]. Nonetheless, random monomer diffusion and rapid polymerization kinetics typically result in highly cross‐linked, low‐crystallinity COF nanofilms. The resulting lack of pore interconnectivity and insufficiently ordered structure pose a substantial challenge to maximizing their molecular‐sieving efficacy [21].

To improve the crystallinity of COF membranes, substantial attempts have focused on regulating monomer diffusivity and/or reactivity via incorporating functional modulators, such as ionic liquids, acids, surfactants, and competitive reactants [22, 23, 24, 25, 26]. For instance, Yang et al. enhanced structural order and separation performance through tannic acid‐mediated heterogeneous nucleation, which provided the kinetic control necessary for ordered stacking and polycrystalline growth [27]. Furthermore, Du et al. pioneered an inorganic ion‐regulated IP modality to refine the crystallization of ionic COFs. By harnessing the “ion pump” effect of strong acid anions to drive interfacial monomer enrichment alongside competitive coordination, this method yielded asymmetric, self‐standing COF films featuring unique Turing structures and optimized transport properties [28]. These additive‐mediated strategies substantially improve the structural order and sieving precision of COF membranes, but are compromised by the unintended introduction of non‐crystalline domains and a surge in synthetic complexity [29, 30]. To address these limitations, we propose a more fundamental strategy: engineering molecular precursors to inherently regulate monomeric diffusivity and reactivity, which are designed to achieve molecular‐level structural control without the complications inherent to external modulators [31, 32].

**FIGURE 1:**
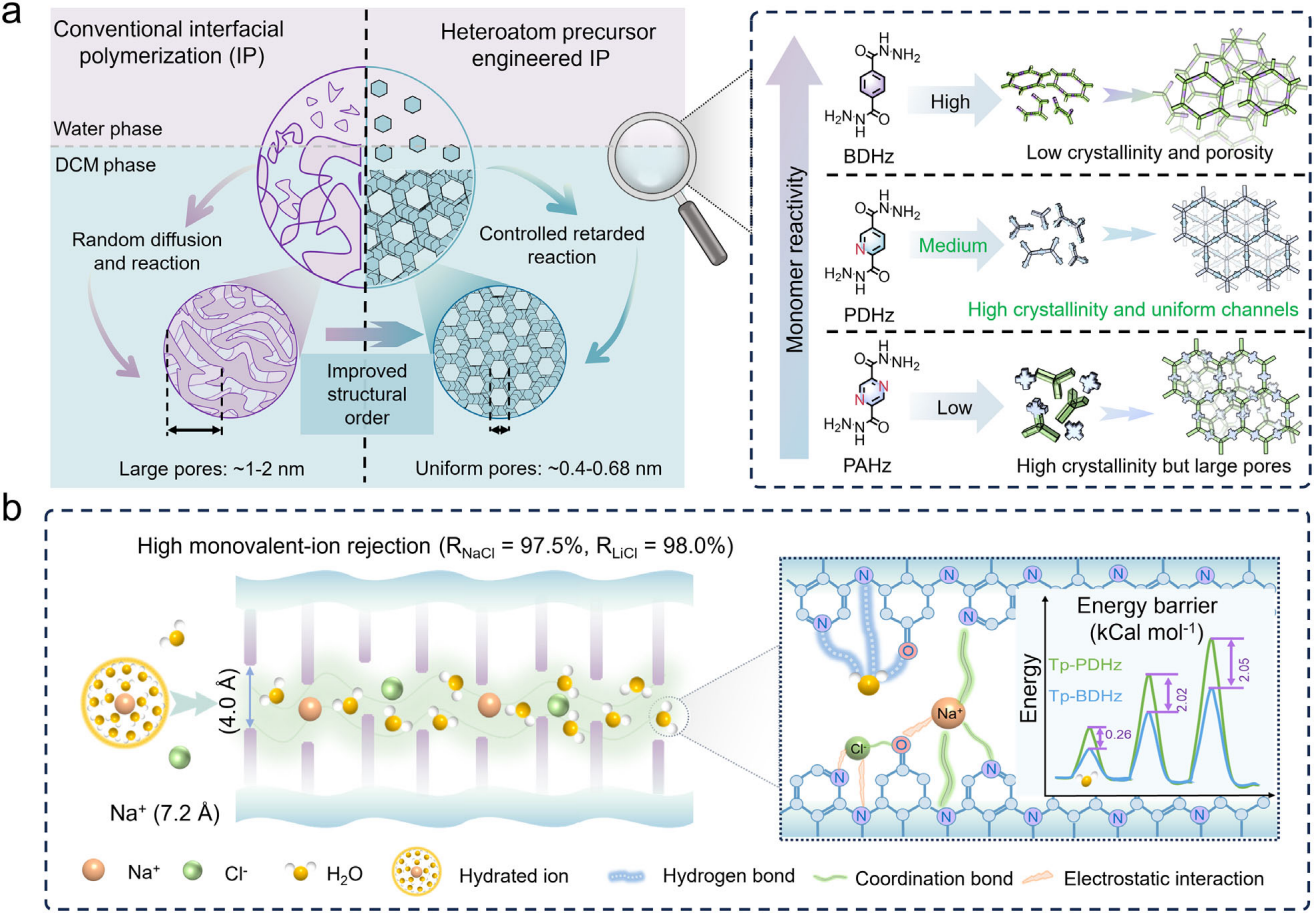
Heteroatom‐engineered COF membranes for water/ion sieving. (a) COF membrane fabrication via interfacial polymerization; (b) Selective transport mechanisms in COF nanochannels.

To this end, we present a heteroatom‐engineered precursor strategy for fabricating crystalline COF membranes via IP reaction optimized for efficient monovalent ion rejection. Specifically, the stepwise introduction of electron‐withdrawing nitrogen atoms into the aromatic rings of dihydrazide monomers enables the systematic tailoring of their electronic and hydrophilic characteristics. This hetero‐nitrogen substitution modification reduces the aromatic electron density, thereby moderating the monomer reactivity while concurrently enhancing hydrophilicity. These modulated properties facilitate the formation of highly ordered, crystalline COF frameworks upon interfacial reaction with Tp, which ultimately dictates the superior desalination performance of the resulting membranes (Figure 1a). Theoretical analyses establish that the synergy between moderate reactivity and enhanced hydrophilicity is fundamental to the formation of crystalline, continuous COF membranes. Leveraging this structure–property correlation, the engineered Tp‐PDHz membranes exhibited exceptional monovalent‑ion rejection (LiCl: 98.0%, NaCl: 97.5%) and a high water permeance of 1.2 L m^−2^ h^−1^ bar^−1^. Molecular dynamics (MD) simulations reveal that the nitrogen‐enriched nanochannels strengthen hydrogen‐bonding networks, thereby accelerating water transport while simultaneously elevating the energy barrier for ion permeation through synergistic electrostatic and van der Waals interactions (Figure 1b). This dual mechanism effectively restricts ion diffusion and enhances salt rejection, demonstrating strong agreement with experimental findings.

## 2 Results and Discussion

### 2.1 Fabrication and Characterization of Free‐Standing COF Nanofilms

**FIGURE 2:**
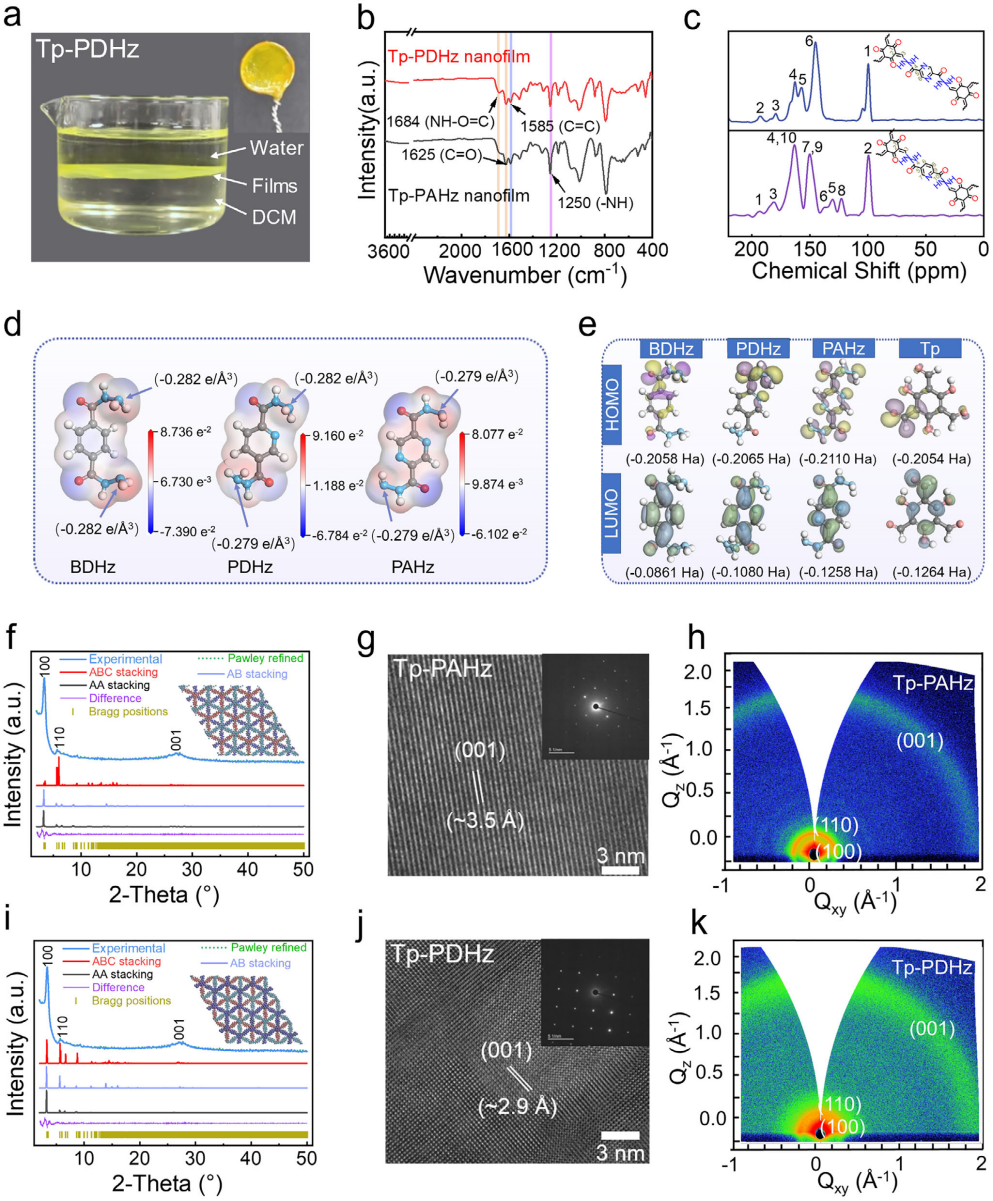
Structural characterization of free‐standing COF nanofilms. (a) The Optical photograph of the Tp‐PDHz freestanding film at the free two‐phase interface (image of the film captured by a wire loop); (b) FT‐IR spectra of Tp‐PDHz and Tp‐PAHz COF nanofilm; (c) Solid‐state ^13^C NMR spectrum of Tp‐PAHz COF (top) and Tp‐PDHz COF (bottom); (d) Electrostatic potential maps of BDHz, PDHz, and PAHz; (e) HOMO and LUMO energy levels of Tp and the hydrazide monomers; PXRD pattern (including experimental, Pawley refined, and simulated PXRD profiles, with the inset showing the COF stacking model diagram), HRTEM image (the lower inset is SAED pattern), and GIWAXS data of Tp‐PAHz COF (f, g, h) and Tp‐PDHz COF (i, j, k).

Initial assessment of film‐forming capacity via support‐free interfacial polymerization (IP) was conducted utilizing 1,3,5‐triformylphloroglucinol (Tp) paired with nitrogen‐varied acylhydrazide precursors (e.g., terephthalohydrazide: BDHz, pyridine‐2,5‐dicarbohydrazide: PDHz, and pyrazine‐2,5‐dicarbohydrazide: PAHz). To overcome the limited solubility of PDHz and PAHz in conventional acetic acid—driven by their reduced basicity and high lattice energy—we employed HCl as the solvent. This strategic substitution yielded completely homogeneous aqueous monomer solutions, a prerequisite for successful COF membrane fabrication. Upon layering the aqueous phase over a dichloromethane (DCM) solution containing Tp monomers, Schiff base condensation was localized at the immiscible interface, generating continuous and mechanically integral nanofilms. The structural integrity of the resulting Tp‐BDHz, Tp‐PDHz, and Tp‐PAHz membranes enabled seamless retrieval using a wire loop (Figure 2a, and Figure S1). The nanofilms were subsequently transferred onto copper grids, porous alumina, and silicon wafers for comprehensive morphological and microstructural characterization.

Fourier transform infrared (FTIR) spectroscopy was employed to characterize the chemical structure of the three COF films. Analysis of their spectra reveals the complete disappearance of the precursor amine (N‐H stretching, 3302 cm^−1^) and the aldehyde (C═O stretching, 1634 cm^−1^) signals. The appearance of a new band at 1582 cm^−1^, assigned to C═C stretching vibrations, signifies the successful formation of β‐ketoenamine tautomeric structure within these three COF films (Figure 2b, Figure S2). Solid‐state ^13^C CP‐MAS NMR spectra (Figure 2c, Figure S3) were further utilized to provide atomic‐scale structural insights into the three COF films. The spectrum of Tp‐BDHz exhibits characteristic resonances at 171 ppm, corresponding to the ketonic C═O, alongside signals at 104 and 163 ppm assigned to the C═C─N linkage. These, coupled with the aromatic and C═O─NH signals between 100–134 ppm, further confirm the formation of the β‐ketoenamine tautomer. Analogous signatures were observed for Tp‐PDHz at 193 ppm (C═O), 99 and 181 ppm (C═C─N), and 163 ppm (C═O─NH), with pyridine carbon signals appearing between 122–150 ppm. Similarly, the Tp‐PAHz displays key resonances at 99 ppm (C═O), 192 and 178 ppm (C═C─N), and 162 ppm (C═O─NH), with additional peaks at 144 and 157 ppm attributed to the pyrazine carbons [33, 34].

The structural evolution of the COF film series, as characterized by scanning electron microscopy (SEM) images, evinces a clear dependence on the nitrogen content of the starting precursors. While Tp‐BDHz produced a continuous surface free of defects, the introduction of more nitrogen atoms in Tp‐PDHz and Tp‐PAHz resulted in localized microcracks and poor continuity, respectively (Figure S4). These morphological transitions were further substantiated by transmission electron microscopy (TEM), with energy‐dispersive X‐ray spectroscopy (EDS) confirming a reduction in elemental homogeneity (C, N, and O) corresponding to the decline in structural integrity (Figures S5–S7). These findings suggest that these morphological differences stem from systematic shifts in acylhydrazide monomer reactivity; specifically, the higher nitrogen density likely reduces their electron density and nucleophilicity, thereby dictating film‐forming kinetics and crystallization pathways during interfacial polymerization [35, 36].

The condensation of Tp with acylhydrazides proceeds via a concerted two‐step mechanism (Figure S8). Following the initial formation of a hydrazone intermediate via nucleophilic attack, the system undergoes irreversible intramolecular tautomerization to produce the fully conjugated β‑ketoenamine linkage. The acidic environment is crucial, as it simultaneously increases the electrophilicity of the aldehyde groups and promotes dehydration. The resulting β‑ketoenamine architecture possesses exceptional chemical stability, anchored by an extensive π‑conjugation system and a stabilizing intramolecular hydrogen‐bonding network. Density functional theory (DFT) calculation was further employed to map their electronic properties (Figure 2d). BDHz displays a higher electron density on its amine nitrogen atoms (−0.282 e/Å^3^) relative to PDHz and PAHz, identifying these amines as the primary nucleophilic centers. The high nucleophilicity of BDHz ensures a robust interfacial reaction with Tp, yielding a more uniform and intact film. In contrast, the increased nitrogen content in PAHz reduces the amine nucleophilicity, thereby slowing the film‐forming kinetics. This kinetic inhibition leads to localized structural failures, accounting for the microcracks observed in the Tp‐PAHz membranes.

DFT calculations were employed to analyze the frontier molecular orbital energy levels of Tp and the three acylhydrazide monomers. Given that the system is determined by a nucleophilic addition–elimination mechanism, we studied the energy alignment between the highest occupied molecular orbitals (HOMO) of the acylhydrazide monomers and the lowest unoccupied molecular orbital (LUMO) of Tp (Figure 2e). The calculations reveal a clear electronic trend as a function of nitrogen content: BDHz possesses a higher HOMO energy level, indicating enhanced electron‐donating capacity; PDHz maintains a moderate HOMO level; and PAHz exhibits a relatively lower HOMO energy. Notably, the calculated HOMO–LUMO energy gaps follow a clear order: BDHz (ΔE = 2.16 eV) < PDHz (ΔE = 2.18 eV) < PAHz (ΔE = 2.28 eV). This trend in energy gaps correlates directly with experimentally observed kinetics; the narrower gap of BDHz facilitates more efficient electron transfer with Tp, yielding the highest reactivity, whereas the wider gap of PAHz results in the most sluggish reaction kinetics. PDHz, possessing an intermediate energy gap, provides a balanced electronic environment conducive to controlled IP reaction [37, 38]. To further probe local reactivity, electrophilic attack indices were computed for the primary amine groups (Figure S9). These results indicate a systematic decline in index values as nitrogen content increases, reflecting a gradual decline in the nucleophilic reactivity of the amino nitrogen sites. This trend is entirely consistent with the frontier orbital analysis, confirming that hetero‐nitrogen incorporation serves as a robust electronic modulator of monomer reactivity [39].

Powder X‐ray diffraction (PXRD) analyses were utilized to elucidate the crystalline structures of COF nanofilms, revealing that heteroatom engineering systematically modulates crystallinity while preserving stacking integrity. The complete absence of monomer‐related diffraction peaks across all specimens confirms the successful formation of distinct crystalline frameworks and excludes the presence of residual physical mixtures (Figure S10). Notably, incorporating heteroatomic nitrogen significantly enhances crystallinity. This improvement is driven by the reduced reactivity of the nitrogen‐substituted hydrazide moieties, which slows the polymerization rate and promotes self‐correction of polymer chains into a more ordered framework. The experimental PXRD patterns of all three films exhibit excellent agreement with simulated profiles based on ABC stacking modes, confirming that each membrane crystallizes in an ABC configuration with precisely modulated unit cell parameters. Tp‐BDHz crystallized in space group R‐3 (a = 30.9414 Å, b = 30.9748 Å, c = 3.0538 Å; α = 90.00°, β = 90.00°, γ = 120.00°; Rwp = 6.70%, Rp = 5.19%), Tp‐PDHz in PM (a = 29.9221 Å, b = 31.0195 Å, c = 3.0838 Å; α = 90°, β = 60°, γ = 90°; Rwp = 17.30%, Rp = 13.2%), and Tp‐PAHz in R‐3 (a = 31.3921 Å, b = 31.3921 Å, c = 3.4160 Å; α = 90°, β = 90°, γ = 120°; Rwp = 13.23%, Rp = 9.82%) (Figure 2f,i, Figures S11–S17).

These crystallographic assignments were substantiated by characteristic diffraction patterns: Tp‐BDHz exhibited peaks at 3.4° (100), 6.0° (110), 9.3° (210), and 26.6° (001). Similarly, Tp‐PDHz showed peaks at 3.3° (100), 5.9° (110), and 27.0° (001), while Tp‐PAHz showed signals at 3.4° (100), 5.8° (110), and 27.1° (001). Complementary high‐resolution TEM images, combined with Fourier filtering analysis, revealed interplanar *d*‐spacings of 0.30 nm (Tp‐BDHz), 0.29 nm (Tp‐PDHz), and 0.35 nm (Tp‐PAHz), correlating precisely with the (001) diffraction planes and providing evidence for the ABC stacking configuration. Sharp selected area electron diffraction (SAED) patterns and continuous crystalline domains spanning expansive membrane regions further evidenced this high crystallinity (Figure 2g,j, and Figures S18 and S19). The anisotropic crystallinity of the film was further elucidated using 2D grazing‑incidence wide‑angle X‑ray scattering (2D‑GIWAXS). The scattering patterns revealed that the heteroatom‑incorporated Tp‑PDHz and Tp‑PAHz COFs possess highly oriented (100) crystal planes and vertically aligned nanochannels—a sharp contrast to the relatively disordered orientation of the Tp‐BDHz analogue (Figure 2h,k, and Figure S20). This structural refinement stems from the attenuated reactivity of the nitrogen‐substituted hydrazide moieties; the decelerated polymerization kinetics promote long‐range crystallographic order, which maximizes channel regularity and suppresses defect formation.

Nitrogen adsorption‐desorption isotherms and Brunauer–Emmett–Teller (BET) analysis were employed to characterize the pore characteristics of three COF films. As illustrated in Figure S21, all three COF variants exhibit a sharp nitrogen uptake at low relative pressures—typical of Type I isotherms—confirming their inherent microporosity [40]. Notably, the BET specific surface area increases significantly with the nitrogen content of the precursors, rising from 27.6 m^2^ g^−1^ for Tp‐BDHz to 460.3 m^2^ g^−1^ for Tp‐PAHz. However, pore size distribution analysis reveals non‐linear variations across the series, with calculated pore widths of 0.64, 0.37, and 0.68 nm for Tp‐BDHz, Tp‐PDHz, and Tp‐PAHz, respectively (Figure S22). The non‐monotonic trends in porosity stem from the balance between polymerization rates and structural order. High‐reactivity monomers lead to rapid, disordered assembly and defective frameworks with low surface areas. In contrast, moderate reactivity allows for ordered growth and minimized pore sizes. As reactivity decreases further, the framework undergoes extended structural equilibration, which maximizes surface area and yields high crystallinity. This state is often characterized by a slight pore enlargement, a signature of framework relaxation during slow growth. These findings highlight that modulating monomer reactivity is essential for tuning the porosity and crystallinity of COF membranes. Thermogravimetric profiles (Figure S 23) demonstrated superior thermal stability across the COF series. Aside from a minor mass loss (∼ 10%) below 100°C —associated with the removal of guests and moisture—the frameworks remain stable up to 320°C. This high decomposition threshold validates the structural resilience of the membranes, confirming their potential for use in thermally intensive environments.

### 2.2 Fabrication and Characterization of COF Membranes

**FIGURE 3:**
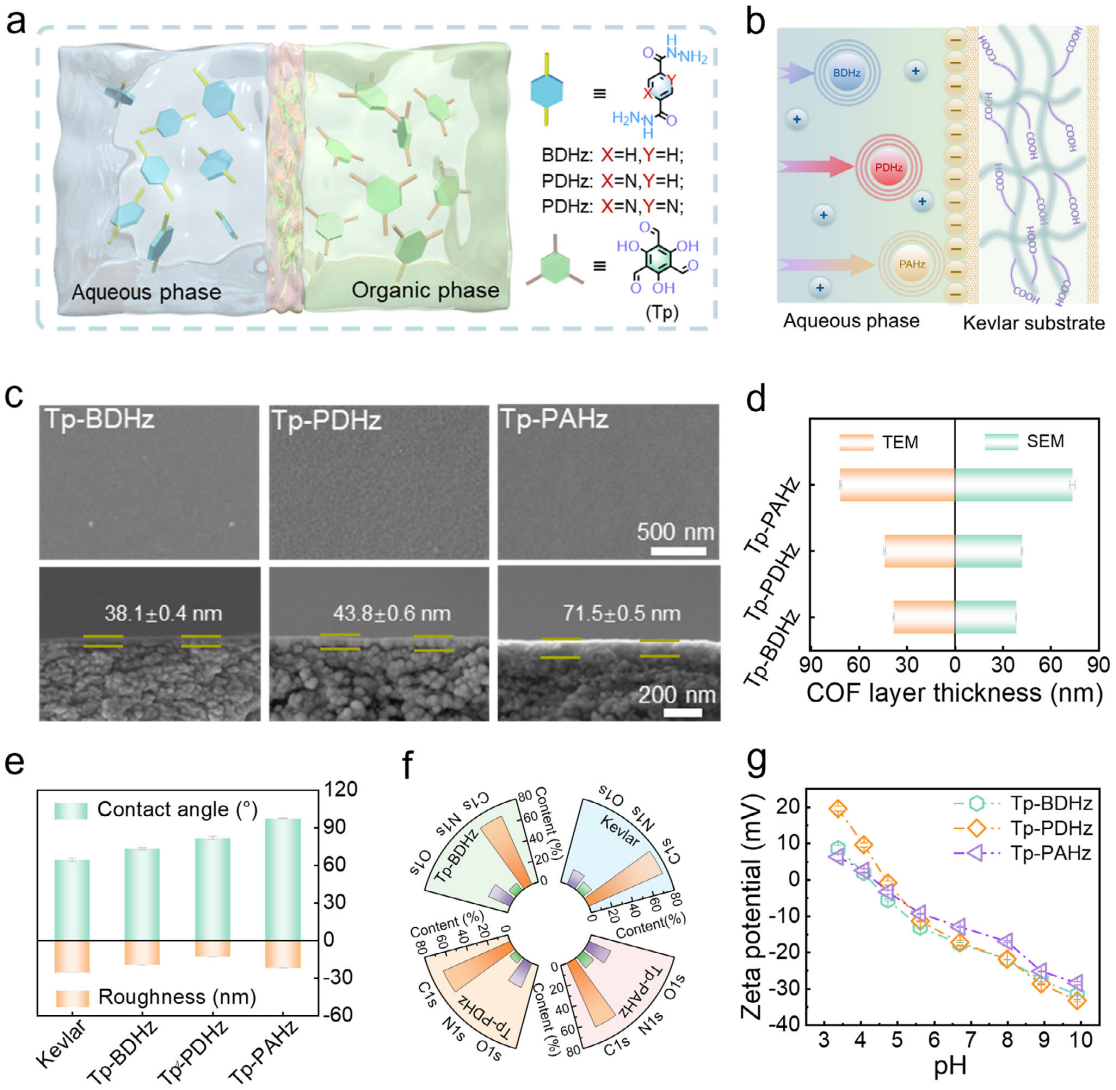
Morphology characterization and physicochemical properties of the membranes. (a) Schematic illustration of counter‑diffusion‑assisted interfacial polymerization for COF membrane fabrication; (b) Schematic illustration of the interactions between the Kevlar substrate and the three hydrazide monomers; (c) Surface SEM (scale bar 500 nm) and cross‐sectional SEM images (scale bar 200 nm) of Tp‐BDHz, Tp‐PDHz and Tp‐PAHz membranes; (d) Comparison of thickness by Cross‐sectional SEM and Cross‐sectional TEM of Tp‐BDHz, Tp‐PDHz and Tp‐PAHz membranes; (e) Comparison of roughness and water contact angle of Tp‐BDHz, Tp‐PDHz and Tp‐PAHz membranes; (f) XPS elemental composition analysis of Kevlar, Tp‐BDHz, Tp‐PDHz, and Tp‐PAHz membranes; (g) Zeta potentials of Tp‐BDHz, Tp‐PDHz and Tp‐PAHz membranes.

Following the successful synthesis of free‐standing COF nanofilms, three COF composite membranes were fabricated via in situ interfacial polymerization onto porous Kevlar substrates using a customized diffusion cell (Figure 3a). The resulting membranes displayed a homogeneous yellow coloration that intensified in proportion to the nitrogen heteroatom content, providing a clear visual distinction from the neutral gray‐white Kevlar support (Figure S24). FT‐IR spectroscopic analysis confirmed that the chemical structures of the composite membranes were identical to those of the freestanding nanofilms. These spectral data collectively validate the successful formation of structurally consistent COF films on the Kevlar substrates (Figure S25).

The Kevlar substrate, derived from poly(p‐phenylene terephthalamide) hydrogel, possesses a surface rich in secondary amine (─NH─) and carboxylic acid (─COOH) moieties. This chemical profile ensures dual compatibility with the COF phase: aromatic backbones facilitate π–π stacking, while functional groups establish extensive hydrogen‐bonding networks. These interactions collectively template the growth of continuous and dense COF layers. To investigate the interactions between hydrazide monomers and the Kevlar substrate, real‐time UV–vis spectroscopy was employed to monitor the concentration dynamics within a biphasic aqueous–organic system (pure DCM as the organic phase, acidic hydrazide solution as the aqueous phase; Figure S26). Results demonstrate that the concentration of the monomers decreases before equilibrating, with the most significant depletion observed in the nitrogen‐rich monomers. This behavior stems from the acidic protonation of the heteroatom sites, which increases the monomeric positive charge density and facilitates their electrostatic attraction to the negatively charged Kevlar. As a result, this enhanced electrostatic interaction contributes to the enrichment of aqueous‐phase monomer within the 3D hydrogel network, subsequently favoring the controlled interfacial polymerization with Tp linkers (Figure 3b) [41]. This substrate‐mediated assembly mechanism effectively reconciles the structural defects previously observed in free‐standing Tp‐PDHz and Tp‐PAHz membranes. The synergistic interfacial interactions between the protonated precursors and the Kevlar support compensate for the inherent limitations of the native crystallization process, contributing to a dual optimization of the COF‐layer structural integrity and its separation performance.

SEM imaging confirmed the formation of three continuous, dense COF selective layers, morphologically distinct from both the porous support and their top‐layer counterparts (Figure 3c). Cross‐sectional SEM analysis revealed significant variations in membrane thickness: Tp‐BDHz (38.1 nm), Tp‐PDHz (43.8 nm), and Tp‐PAHz (71.5 nm), results which were further validated by TEM cross‐sections (Figure 3d, Figure S27). The higher reactivity of BDHz monomers substantially facilitates the rapid formation of an initially compact film barrier, which effectively suppresses subsequent monomer diffusion and this leads to a thinner COF layer on the hydrogel support. AFM characterization illustrated a significant reduction in surface roughness relative to the Kevlar substrate (*Ra* = 25.5 nm), with values of 19.5, 13.0, and 21.8 nm for Tp‐BDHz, Tp‐PDHz, and Tp‐PAHz, respectively (Figures S28 and S29). These metrics indicate the formation of smooth, compact surface topographies. Furthermore, water contact angle (WCA) measurements indicated a progressive increase in hydrophobicity across the COF series in correlation with nitrogen content (Figure 3e). This trend is likely driven by two synergistic factors: first, the deposition of low‐polarity aromatic selective layers lowers the surface energy of the Kevlar substrate; second, the specific chemical architectures of the various COFs modulate the intrinsic wettability of the composite membrane [42, 43].

A systematic investigation into the impact of monomer concentration and reaction kinetics revealed significant regulatory patterns governing COF membrane assembly. SEM imaging demonstrated that rising the concentration of either the acylhydrazide or the Tp substantially enhanced nucleation density, resulting in increased structural compactness (Figures S30–S35). Higher monomer concentrations accelerate polymerization kinetics, favoring heterogeneous nucleation; this increases the density of polymerization nuclei, which subsequently coalesce into continuous, dense surface architectures. Real‐time monitoring across all three COF variants on Kevlar substrates confirmed the progressive coverage of substrate pores and the eventual formation of a cohesive selective layer as reaction time increased (Figures S36–S38). Prolonged reaction duration not only facilitates continuous COF growth but also promotes framework self‐healing, ultimately yielding defect‐free COF layers with long‐range structural order.

To confirm the chemical compositions of the as‐synthesized membranes, we performed a systematic XPS analysis of the Kevlar substrate and the three COF derivatives (Figure 3f, Table S1). All TFC membranes exhibited distinct compositional shifts—specifically reduced carbon content and elevated oxygen/nitrogen levels relative to the pristine substrate—validating the successful formation of COF layers. High‐resolution deconvolution of the C 1s, N 1s, and O 1s core‐level spectra consistently confirmed the formation of β‐ketoenamine linkages across all three COF structures, showing excellent correlation with complementary FT‐IR and solid‐state ^13^C NMR analyses (Figures S39–S42). The N 1s spectra provided evidence of chemical differentiation: Tp‐PDHz exhibited a characteristic pyridinic nitrogen peak at 401.9 eV, whereas Tp‐PAHz displayed a distinct pyrazinic nitrogen signature at 399.9 eV, indicate of successful heteroatom incorporation. Zeta potential measurements revealed isoelectric points (IEPs) near pH ≈ 4 for all three COF membranes, for all three COF variants, indicating consistently negative surface charges under neutral conditions (Figure 3g). This electronegative character is predominantly governed by the abundant carboxylic acid functionalities of the underlying Kevlar substrate, which exert a defining influence on the overall surface properties of the composite membranes [44, 45].

### 2.3 Desalination Performance of COF Membranes

**FIGURE 4:**
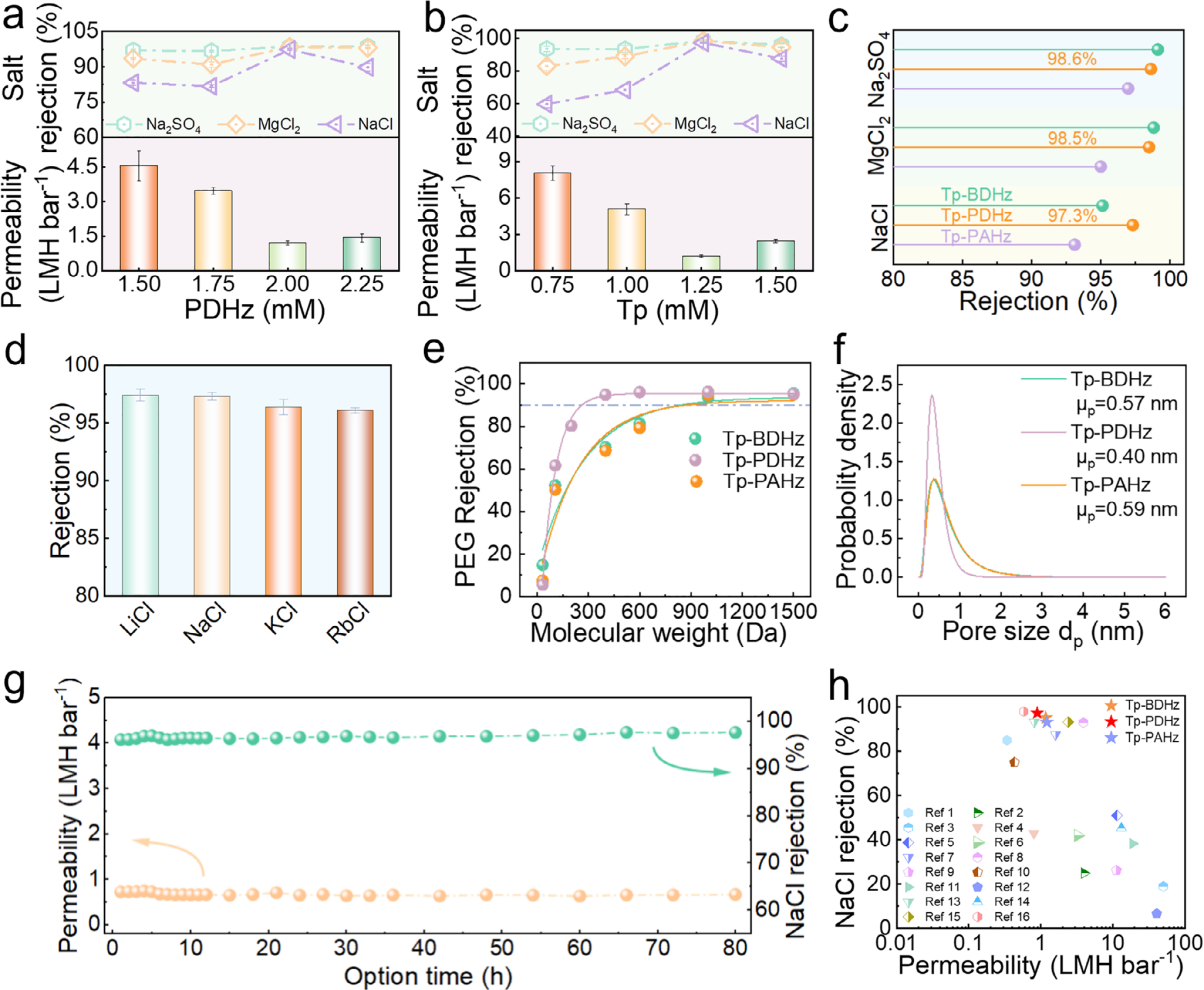
Performance and application of Tp‐BDHz, Tp‐PDHz and Tp‐PAHz COF membranes. (a, b) Water permeance and salt rejection of Tp‐PDHz COF membranes under different PDHz content and Tp content; (c) Comparison of salt rejection Rates for Tp‐BDHz, Tp‐PDHz, and Tp‐PAHz COF Membranes (d) Rejection of four monovalent salts across the Tp‐PDHz COF membranes; error bars represent the standard deviation (SD) derived from at least three independent membrane samples (n = 3) synthesized from separate batches. (e) Molecular‐weight‐dependent rejection of uncharged organic solutes (feed concentration = 1000 ppm); (f) The pore size distributions determined from solute rejection data; (g) Long‐term operational stability performance of Tp‐PDHz membrane; (h) Membranes performance in this work compared to other membranes in the literature: Ref 1 (Graphene/GO) [46]; Ref 2 (Laminated GO) [47]; Ref 3 (GO/TMC) [48]; Ref 4 (GO/PECs) [49]; Ref 5 (Graphene/CNT) [50]; Ref 6 (Graphene) [51]; Ref 7 (MoS_2_) [52]; Ref 8 (COF‐DhTG_Cl_) [53]; Ref 9 (TpPa) [54]; Ref 10 (TpPa‐SO_3_H) [55]; Ref 11 (TpPa) [56]; Ref 12 (TpHz) [57]; Ref 13 (PaTP_0.05%_−TMC_0.2%/30_) [58]: Ref 14 (TpPa–SO_3_H/TpPa–SO_3_H/MPAN) [59]; Ref 15 (PES‐(TMC‐MPD)) [60]; Ref 16 (PK/PSf‐(TMC‐MPD)) [61].

A systematic investigation into the desalination performance of COF membranes identifies a definitive correlation between monomer stoichiometry and desalination efficiency. Under standardized testing conditions (2000 ppm NaCl, 15 bar, 25°C), altering the PDHz concentration (1.5–2.25 mM) at a fixed Tp concentration (1.25 mM) revealed a distinct, non‐linear response in membrane performance: water flux exhibited a parabolic trend, whereas the rejection of Na_2_SO_4_, MgCl_2_, and NaCl concurrently peaked before declining. The optimal performance emerged consistently at 2.0 mM PDHz, yielding an exceptional balance between water permeability (1.2 L m^−2^ h^−1^ bar^−1^) and superior salt rejection (98.6% Na_2_SO_4_, 98.5% MgCl_2_, 97.5% NaCl) (Figure 4a). Microstructural analysis elucidates the mechanistic basis of this nonlinear performance trend. At the stoichiometric optimum, retarded polymerization kinetics favor the formation of highly ordered and defect‐free film architectures responsible for improved monovalent‐ion‐sieving characteristics. Deviations from this stoichiometric balance induces structural defects: lower concentrations result in incomplete monomer assembly, while excessive concentrations trigger chaotic polymerization with macroscopic void formation, thereby inevitably compromising the desalination efficacy. This optimization principle was validated by altering Tp concentrations (0.75–1.5 mM) at a fixed 2.0 mM of PDHz concentration confirmed a consistent performance peak (Figure 4b). Significantly, the optimal ratio (1.25 mM Tp to 2.0 mM acylhydrazide) exhibited remarkable universality across all heteroatom‐engineered variants, establishing precise monomer stoichiometry as a fundamental design parameter for maximizing desalination performance (Figures S43–S47).

Comparative analyses of the three COF membranes fabricated at identical monomer concentrations reveal distinct transport characteristics. The Tp‐BDHz, Tp‐PDHz, and Tp‐PAHz membranes exhibited water permeance of 1.1, 1.2, and 1.3 L m^−2^ h^−1^ bar^−1^, paried with corresponding NaCl rejection of 95.1%, 97.5%, and 93.1%, respectively (Figure 4c). These performance variations are primarily attributed to the different reactivities of the acylhydrazide monomers, which dictate the resulting pore width, channel ordering, and transport pathways. The slight increase in water permeance from Tp‐BDHz (1.1 L m^−2^ h^−1^ bar^−1^) to Tp‐PDHz (1.2 L m^−2^ h^−1^ bar^−1^) and Tp‐PAHz (1.3 L m^−2^ h^−1^ bar^−1^) stems from a balance between competing structural factors. While Tp‐PAHz possesses the largest pore size (∼0.68 nm) to reduce intrinsic transport resistance, its greater thickness (71.5 nm) introduces additional diffusional resistance. Conversely, despite its smaller pore size (∼0.37 nm), Tp‐PDHz benefits from highly ordered, vertically aligned nanochannels that provide direct transport pathways with reduced tortuosity, thereby compensating for its smaller pore dimensions. Consequently, the moderate thickness (43.8 nm) and highly ordered pore architecture of Tp‐PDHz collectively achieve optimized transport efficiency.

In view of ion separation, the moderate reactivity of PDHz facilitates controlled polymerization, yielding a framework with an optimized effective pore size (∼0.37 nm) that maximizes desalination efficacy. In contrast, the high reactivity of BDHz induces rapid, disordered assembly—giving rise to structural defects and nonuniform pores (∼0.64 nm)—while the low reactivity of PAHz results in framework relaxation and pore enlargement (∼0.68 nm). Beyond steric effects, the abundant nitrogen‐rich sites within Tp‐PDHz enhance the exclusion of sodium ions through localized electrostatic repulsion. Consequently, Tp‐PDHz achieves superior selective transport via a synergy of steric pore confinement and nitrogen‐mediated interactions, whereas its analogues underperform due to suboptimal structural and chemical configurations.

Further evaluation of the Tp‐PDHz membrane against other monovalent ion species demonstrated high rejections of 98.0% for LiCl, 96.3% for KCl, and 96.1% for RbCl (Figure 4d). Moreover, the membrane exhibited excellent rejection toward divalent salts, with values of 99.1% for CaCl_2_, 99.0% for NiCl_2_, 98.5% for MgSO_4_, 95.6% for MnCl_2_, and 93.5% for CuCl_2_ (Figure S48). These robust rejection profiles across both mono‐ and divalent species arise from the synergy of strict steric exclusion within the ultramicroporous channels (∼0.40 nm) and enhanced electrostatic repulsion from the nitrogen‐enriched pore walls. These findings validate this heteroatom‐engineering approach as a universal design principle, offering a scalable strategy for developing high‐performance membranes with broad‐spectrum ion selectivity. Beyond the intrinsic COF channel properties, the choice of the underlying support proved critical to achieving these superior separations. Control experiments using PES, PVDF, and PAN substrates under identical IP conditions failed to yield uniform COF layers, resulting in distinctly lower NaCl rejection and underscoring the unique advantage of the Kevlar support (Figures S49 and S50). The aromatic backbone and abundant polar amide groups (─NH─ and ─COOH) of Kevlar enable effective anchoring of the COF layer via π–π stacking and hydrogen‐bonding interactions, enabling the formation of a continuous, defect‐free membrane.

To characterize the pore properties of COF membranes under aqueous conditions, molecular weight cut‐off (MWCO) measurements were performed utilizing neutral polyethylene glycol (PEG) solutes as molecular probes. The Tp‐BDHz, Tp‐PDHz, and Tp‐PAHz membranes displayed MWCO values of 840, 269, and 850 Da, which correspond to calculated effective pore diameters of 0.57, 0.40, and 0.59 nm, respectively (Figure 4e,f). The pore size distributions derived from these rejection profiles showed high consistency with BET characterization, validating the methodological reliability. The more sharpened pore size distribution of Tp‐PAHz confirms the high effectiveness of utilizing hetero‐nitrogen precursor engineering in regulating the IP process, yielding structurally ordered, ultra‐microporous COF membranes. As shown in Figure 4g, the Tp‐PDHz membrane exhibited robust durability during an 80‐hour continuous operation run at 15 bar, maintaining a steady water flux and consistently achieving NaCl rejection exceeding 97.5%. This sustained performance underscores the mechanical and chemical resilience of Tp‐PDHz membranes under extended operational stress. Additional tests at varying pressures (5–20 bar) further confirmed the membrane's structural stability, with linear flux–pressure behavior and stable NaCl rejection (> 97.5%) across all tested conditions (Figures S51and S52). Furthermore, a comparative assessment (Figure 4h, Table S2) positions these COF membranes at the forefront of the field, especially surpassing advanced COF membranes. These findings validate the heteroatom precursor engineering strategy, demonstrating that precise molecular design can simultaneously optimize separation efficiency and structural integrity, thereby providing a definitive blueprint for next‐generation high‐performance desalination technologies.

### 2.4 Mechanisms of Heteroatom‑Engineered Interfacial Polymerization

**FIGURE 5:**
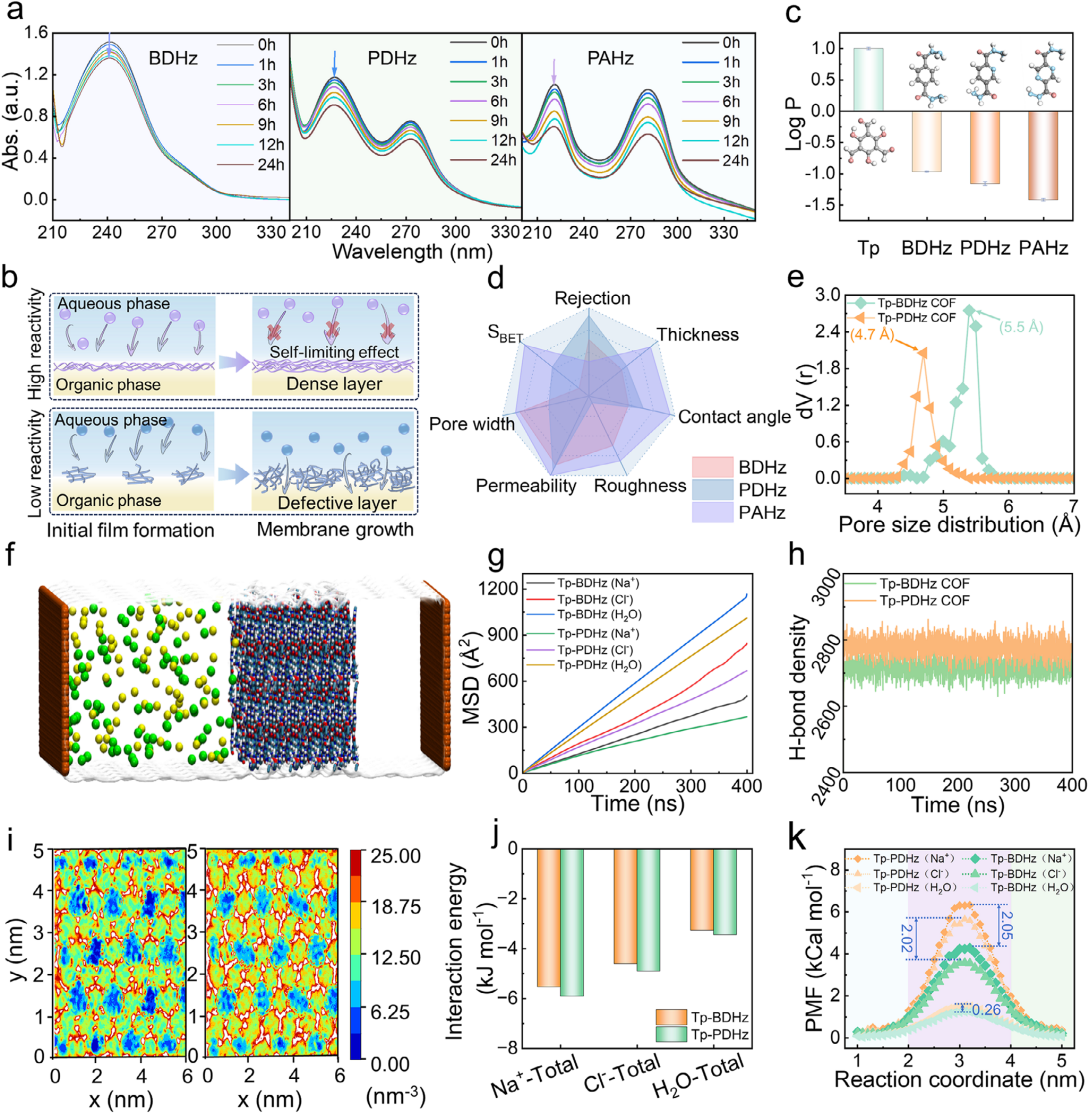
Mechanisms of interfacial polymerization and molecular‐scale transport in COF membranes. (a) Time‐dependent UV–vis absorption spectra of BDHz, PDHz, and PAHz in the aqueous phase; (b) Schematic of self‑limiting interfacial polymerization in COF membrane formation; (c) Oil–water partition coefficients of Tp and hydrazide monomers (BDHz, PDHz, PAHz); error bars represent the standard deviation (SD) derived from at least three independent experiments (n = 3). (d) Radar chart comparing the physicochemical properties of Tp‐BDHz, Tp‐PDHz, and Tp‐PAHz COF membranes; (e) Simulated pore size distribution of Tp‐BDHz and Tp‐PDHz; (f) Simulation setup for Tp‐PDHz membranes, featuring a five‐layer COF structure sandwiched between graphene pistons (orange) under a 15 bar pressure gradient, with water molecules shown in light blue; (g) Mean squared displacement (MSD) of H_2_O, Na^+^, and Cl^−^ within the membrane pores; (h) Number of hydrogen bonds formed between each COF membrane and water molecules. (i) 2D density distribution of water molecules within the COF pores (Tp‐BDHz, left; Tp‐PDHz, right); (j) Interaction energies between the COF membranes and H_2_O/ Na^+^/ Cl^−^ species; (k) Potential of mean force (PMF) profiles for H_2_O, Na^+^, and Cl^−^ transport across the COF membranes.

To systematically monitor monomer dissipation kinetics, UV–vis spectroscopy was used during the interfacial polymerization for fabricating the COF membranes. Time‐dependent sampling and spectral analysis of the organic and aqueous phases precisely tracked the dynamic monomer depletion profiles (Figure 5a, Figure S53). Quantitative kinetic analysis of the aqueous‐phase hydrazide concentration reveals distinct stages: during the initial film‐formation phase, the fitted depletion rates for BDHz, PDHz, and PAHz are 24.72 × 10^−3^, 23.55 × 10^−3^, and 18.34 × 10^−3^ mmol h^−1^, respectively. However, in the subsequent film‐growth stage, these rates reversed to 2.74 × 10^−3^, 7.62 × 10^−3^, and 22.18 × 10^−3^ mmol h^−1^ (Figure S54). These data provide clear evidence of the self‐limiting effect inherent to high‐reactivity monomers during interfacial polymerization. In this context, the formation process is divided into two distinct phases: initial film formation and subsequent growth. While BDHz forms a dense, self‐limiting initial layer that arrests further growth, PAHz yields a defective initial architecture that allows continuous monomer flux to the reaction zone, resulting in a significantly thicker film (Figure 5b). Notably, PDHz exhibits a balanced growth profile; minor initial defects are mitigated through structural self‐correction, resulting in a robust membrane of moderate thickness.

The oil–water partition coefficients (Log *P*) analyses reveal a clear physicochemical gradient across the monomer series (Figure 5c). While Tp remains hydrophobic (Log *P* = 1.0), the acylhydrazide monomers demonstrate increasing hydrophilicity with higher nitrogen content (BDHz: −0.97; PDHz: −1.16; PAHz: −1.44). This trend is attributed to the enhanced capacity for hydrogen‐bond formation, which strengthens the hydration effect of the monomers. Increasing nitrogen atom content strengthens hydrogen‐bond formation with water molecules, resulting in progressively more pronounced hydration effects [62, 63]. Consequently, the intensified hydration limits the diffusion of acylhydrazides across the aqueous–organic interface, preventing the rapid assembly of dense COF architectures. These differences in diffusion kinetics ultimately account for the varying degrees of structural integrity observed in the resulting COF layers.

A systematic evaluation of monomer partition coefficients, substrate–interface interactions, and intrinsic reactivity identifies the PDHz‐Tp system as the optimal configuration for fabricating dense COF membranes via interfacial polymerization (Figure 5d). First, the balanced partition coefficient of PDHz establishes a favorable concentration gradient at the interface, facilitating the formation of a uniform and continuous primary layer. Second, the moderate positive charge of PDHz under acidic conditions elicits optimal electrostatic attraction toward the negatively charged Kevlar substrate, promoting uniform growth while mitigating the particle aggregation. More importantly, the moderate reactivity of PDHz facilitates sufficient self‐correction of polymeric chains into ordered, crystalline frameworks with narrowed pore size distribution. This multi‐parameter synergy harmonizes monomer transport, interfacial energy, and reaction kinetics, enabling the construction of a well‐oriented ultramicroporous COF separation layer (Figure S55).

### 2.5 Molecular‐Scale Transport in Heteroatom‑Engineered COF Membranes

To elucidate the mechanistic role of heteroatom engineering in water transport and monovalent‐ion rejection, MD simulations were performed on Tp‐BDHz and Tp‐PDHz membranes. The simulation system, comprising a five‐layer COF assembly and pressure‐regulated graphene pistons, was subjected to a 15‐bar differential to characterize transmembrane transport. Results indicate that the introduction of varying nitrogen content preserves the integrity of water‐transport pathways across both systems. However, the pore‐size distribution analysis (Figure 5e), highlights the structural divergence caused by heteroatom engineering, yielding effective pore diameters of 4.7 Å (Tp‐BDHz) and 5.5 Å (Tp‐PDHz), which likely governs the observed ion‐rejection efficiency. While both COFs adopt an ABC stacking mode, nitrogen heteroatom engineering in Tp‐PDHz results in a more ordered framework compared to the distorted geometry of Tp‐BDHz, reflecting the difference in COF pore sizes. During a 400 ns desalination simulation, both membranes exhibited high water permeability and significantly restricted ion transport, confirming their viability for desalination applications (Figure 5f, Figures S56–S59). Specifically, the enhanced ion‐sieving performance of Tp‐PDHz is specifically driven by its more ordered architecture and narrower pore size.

To elucidate the underlying separation mechanism, we examined the intrapore diffusion behavior of water and ions. Mean squared displacement (MSD) analysis (Figure 5g) revealed that water molecules possess substantially higher mobility than Na^+^ and Cl^−^ ions across both COF layers. The diffusion coefficients for water were calculated at 4.75 × 10^−6^ cm^2^ s^−1^ in Tp‐BDHz and 4.18 × 10^−6^ cm^2^ s^−1^ in Tp‐PDHz. In contrast, ionic mobility was markedly constrained, with Na^+^ coefficients of 2.06 × 10^−6^ and 1.47 × 10^−6^ cm^2^ s^−1^ for Tp‐BDHz and Tp‐PDHz, respectively. This transport disparity originates from nitrogen‐directed modulation of the hydrazide monomers. The enriched nitrogen content in Tp‐PDHz optimizes the localized charge distribution and hydrogen‐bonding networks, effectively trapping ions through electrostatic interactions while maintaining directional water permeability. Conversely, the lower nitrogen content in Tp‐BDHz offers less resistance to ion mobility, highlighting the critical role of heteroatom density in enhancing the water‐ion selectivity. Hydrogen bond quantification (Figure 5h) supports this interpretation, confirming significantly more extensive hydration within the Tp‐PDHz framework. Furthermore, 2D water density distributions (Figure 5i) reveal denser aqueous layering within these pores. This suggests that a robust hydration network effectively reduces the COF pore size, favoring the enhanced rejection of small monovalent ions via steric hindrance.

Interaction energy analysis reveals that both COF frameworks exhibit higher binding affinities for Na^+^ and Cl^−^ relative to water molecules, suggesting an inherent thermodynamic preference for water transport (Figure 5j). Energy decomposition confirms that electrostatic interactions identify electrostatic interactions as the dominant factor governing membrane–ion interaction. To elucidate the kinetics of this process, we performed umbrella sampling to determine the energy barriers associated with transmembrane transport (Figure 5k, Figures S60–S62). These calculations reveal that Na^+^ and Cl^−^ encounter significantly greater energy barriers than water, consistent with the high‐precision water‐ion separation. Specifically, Tp‐PDHz demonstrates significantly higher energy barriers for Na^+^ (2.05 kcal mol^−1^) and Cl^−^ (2.024 kcal mol^−1^) compared to Tp‐BDHz, accounting for its superior salt rejection performance. These energetic profiles confirm that nitrogen‐based heteroatom engineering is the primary driver of enhanced ionic selectivity. Meanwhile, the marginally lower water transport energy barrier in Tp‐PDHz (a 0.26 kcal·mol^−1^ differential) aligns with the subtle variations in water flux between the two membranes. Overall, these results demonstrate that heteroatom precursor engineering allows for the simultaneous optimization of permeability and selectivity by precisely refining both the pore architecture and the internal channel microenvironment.

## 3 Conclusion

This study establishes heteroatom precursor engineering as a robust molecular‐level strategy to fabricate nitrogen‐rich COF membranes for improved water desalination performance. The strategic substitution of nitrogen within the aromatic rings of hydrazide monomers enables a gradient reduction of both their oil–water partition coefficients and chemical reactivity, thereby effectively moderating the interfacial reaction kinetics. This inhibited diffusion–reaction behavior facilitates the self‐healing of nascent polymeric chains into highly ordered networked structures, yielding crystalline nanofilms with precisely controlled, ultrafine pore widths (0.40–0.68 nm). The optimized Tp‑PDHz membrane demonstrates exceptional monovalent‑ion rejection (NaCl: 97.5%; LiCl: 98.0%) coupled with a high water permeance of 1.2 L m^−2^ h^−1^ bar^−1^. Experimental and simulation results reveal that this performance arises from nitrogen‐enriched nanochannels that facilitate ordered hydration pathways while simultaneously elevating the energetic barriers for ion transport. Ultimately, these findings provide a rational molecular roadmap for the design of next‐generation COF membranes with precisely tailored transport functions for advanced desalination and ion separation.

## 4 Experimental Section/Methods

### 4.1 Materials and Methods

Kevlar aramid fibers (linear density: 220 dtex; break strength: 49 N; modulus: 3.000 cN/tex) were sourced from Thread Exchange (Delaware, USA). Novatexx 2471 non‐woven fabric was purchased from Freudenberg Performance Materials (Weinheim, Germany). Tp (>98.0%) and Terephthalic Dihydrazide (BDHz) (>97.0%) were acquired from the Shanghai Tensus Bio‐tech Co., Ltd. Dimethyl pyridine‐2,5‐dicarboxylate (>97.0%) and Pyrazine‐2,5‐dicarboxylic acid dihydrate (>97.0%) were purchased from Leyan (Cat No.1062335, Cat No.1199388; leyan, Shanghai, China). Potassium hydroxide (99.0%), n‐hexane (>99.0%), dichloromethane (DCM), acetone, tetrahydrofuran (THF), hydrazinium hydroxide solution (80%), thionyl chloride (99.0%), methanol, ethanol, and dimethyl sulfoxide (DMSO, 99.5%) were obtained from Sinopharm Chemical Reagent (Shanghai, China). A range of polyethylene glycol (PEG, AR, Merck) with molecular weights of 106, 400, 600, 1000, and 1500 Da was purchased from Sinopharm Chemical Reagent (Shanghai, China). Deionized water purified by a Milli‐Q ultrapure system was used throughout this work.

### 4.2 Fabrication of COF Free‐Standing Nanofilm and Membrane

In situ interfacial Schiff‑base polymerization was employed to fabricate Tp‑PDHz thin films on Kevlar hydrogel supports. The organic phase was prepared by dissolving Tp (13.13 mg, 1.25 mM) in dichloromethane (50 mL). The aqueous phase contained PDHz (19.52 mg, 2.0 mM) and HCl (125 µL) in water (50 mL). A Kevlar membrane (4.5 cm in diameter) was vertically fixed in a U‑shaped diffusion cell, separating it into two 30 mL chambers. The organic and aqueous phases were introduced into the opposing chambers. The sealed system was maintained at 28°C and 30% relative humidity for 24 h. After polymerization, the resulting composite membrane was thoroughly rinsed with water, soaked in ultrapure water, and subsequently characterized. The Tp‑BDHz and Tp‑PAHz membranes were synthesized following an identical procedure, with BDHz (19.41 mg) or PAHz (19.62 mg) used in the aqueous phase, respectively.

## Author Contributions

**Muning Chen**: conceptualization, formal analysis. **Junyong Zhu**: conceptualization, methodology, funding acquisition, supervision, writing – review and editing. **Yatao Zhang**: supervision, resources, writing – review and editing, funding acquisition. **Guishan Hu**: conceptualization, methodology, formal analysis, investigation, project administration, data curation, writing – original draft. **Yu Zhen**: data curation, investigation, formal analysis. **Miaomiao Tian**: conceptualization, methodology, software, formal analysis, supervision. **Jingwei Hou**: writing – review and editing, validation, methodology, conceptualization. **Yong Wang**: conceptualization, writing – review and editing, visualization, supervision, formal analysis.

## Funding

Major Research Plan of the National Natural Science Foundation of China (92475120); the National Natural Science Foundation of China (52573238); the Outstanding Youth Fund of Henan Scientific Committee (252300421182); and the Science and Technology Innovation Leading Talent Support Program of Henan Province (254200510023).

## Conflicts of Interest

The authors declare no conflicts of interest.

## Supporting information




**Supporting File**: advs77400‐sup‐0001‐SuppMat.docx.

## Data Availability

The data that support the findings of this study are available from the corresponding author upon reasonable request.

## References

[1] W. Liu , Z. Fu , M. T. H. van Vliet , et al., “Global Overlooked Multidimensional Water Scarcity,” Proceedings of the National Academy of Sciences 122, no. 26 (2025): 2413541122, 10.1073/pnas.2413541122. PMC12232667 40549919

[2] L. Rosa , D. D. Chiarelli , M. C. Rulli , J. Dell'Angelo , and P. D'Odorico , “Global Agricultural Economic Water Scarcity,” Science Advances 6, no. 18 (2020): aaz6031, 10.1126/sciadv.aaz6031. PMC7190309 32494678

[3] V. G. Gude , “Desalination and Water Reuse to Address Global Water Scarcity,” Reviews in Environmental Science and Bio/Technology 16, no. 4 (2017): 591–609, 10.1007/s11157-017-9449-7.

[4] C. He , Z. Liu , J. Wu , et al., “Future Global Urban Water Scarcity and Potential Solutions,” Nature Communications 12, no. 1 (2021): 4667, 10.1038/s41467-021-25026-3. PMC8333427 34344898

[5] A. Pistocchi , T. Bleninger , C. Breyer , et al., “Can Seawater Desalination be a Win‐Win Fix to Our Water Cycle?,” Water Research 182 (2020): 115906, 10.1016/j.watres.2020.115906. 32629317 PMC7487278

[6] A. Ilyas and I. F. J. Vankelecom , “Designing Sustainable Membrane‐Based Water Treatment via Fouling Control Through Membrane Interface Engineering and Process Developments,” Advances in Colloid and Interface Science 312 (2023): 102834, 10.1016/j.cis.2023.102834. 36634445

[7] J. Zhu , S. Yuan , J. Wang , Y. Zhang , M. Tian , and B. Van der Bruggen , “Microporous Organic Polymer‐Based Membranes for Ultrafast Molecular Separations,” Progress in Polymer Science 110 (2020): 101308, 10.1016/j.progpolymsci.2020.101308.

[8] X. Feng , J. Zhu , J. Jin , Y. Wang , Y. Zhang , and B. Van der Bruggen , “Polymers of Intrinsic Microporosity for Membrane‐Based Precise Separations,” Progress in Materials Science 144 (2024): 101285, 10.1016/j.pmatsci.2024.101285.

[9] G. Hu , B. Zhou , Y. Zhen , et al., “Covalent Organic Framework Catalytic Membranes for Durable Multitasking Water Purification,” Chemical Science 16, no. 39 (2025): 18141–18151, 10.1039/D5SC04204F. 40936606 PMC12421428

[10] M. B. Asif , S. Kim , T. S. Nguyen , J. Mahmood , and C. T. Yavuz , “Covalent Organic Framework Membranes and Water Treatment,” Journal of the American Chemical Society 146, no. 6 (2024): 3567–3584, 10.1021/jacs.3c10832. 38300989 PMC10870710

[11] S. Yuan , X. Li , J. Zhu , G. Zhang , P. Van Puyvelde , and B. Van der Bruggen , “Covalent Organic Frameworks for Membrane Separation,” Chemical Society Reviews 48, no. 10 (2019): 2665–2681, 10.1039/C8CS00919H. 31025660

[12] A. Jrad , M. A. Olson , and A. Trabolsi , “Molecular Design of Covalent Organic Frameworks for Seawater Desalination: A State‐Of‐The‐Art Review,” Chemistry 9, no. 6 (2023): 1413–1451, 10.1016/j.chempr.2023.04.012.

[13] T. H. Lee , M. Balcik , Z. Ali , et al., “Microporous Polyimine Membranes for Efficient Separation of Liquid Hydrocarbon Mixtures,” Science 388, no. 6749 (2025): 839–844, 10.1126/science.adv6886. 40403073

[14] R. Shevate and D. L. Shaffer , “Large‐Area 2D Covalent Organic Framework Membranes With Tunable Single‐Digit Nanopores for Predictable Mass Transport,” ACS Nano 16, no. 2 (2022): 2407–2418, 10.1021/acsnano.1c08804. 35135189

[15] A. Nowbahar , V. Mansard , J. M. Mecca , M. Paul , T. Arrowood , and T. M. Squires , “Measuring Interfacial Polymerization Kinetics Using Microfluidic Interferometry,” Journal of the American Chemical Society 140, no. 9 (2018): 3173–3176, 10.1021/jacs.7b12121. 29432004

[16] H. Zhang , Q. He , J. Luo , Y. Wan , and S. B. Darling , “Sharpening Nanofiltration: Strategies for Enhanced Membrane Selectivity,” ACS Applied Materials & Interfaces 12, no. 36 (2020): 39948–39966, 10.1021/acsami.0c11136. 32805813

[17] V. Freger and S. Srebnik , “Mathematical Model of Charge and Density Distributions in Interfacial Polymerization of Thin Films,” Journal of Applied Polymer Science 88, no. 5 (2003): 1162–1169, 10.1002/app.11716.

[18] X. Sun , M. Di , L. Gao , et al., “Scalable Fabrication of Integrated Covalent Organic Framework Membrane With Selective Ion Transport for Efficient Salinity Gradient Energy Harvesting,” Nano Energy 128 (2024): 109959, 10.1016/j.nanoen.2024.109959.

[19] X.‐F. Hu , T. Jiang , H. Fan , et al., “Dual‐Regulated Covalent Organic Framework Membranes With Near‐Theoretical Pore Sizes for Angstrom‐Scale Ion Separations,” Science Advances 11, no. 46 (2025): ady3587, 10.1126/sciadv.ady3587. PMC12609051 41223271

[20] J. Yuan , Z. Mai , M. Parkinson , et al., “Precision‐Engineered Crystalline Covalent Organic Framework Membranes With Staggered ABC Stacking for High‐Performance Desalination,” Journal of the American Chemical Society 147, no. 51 (2025): 47477–47488, 10.1021/jacs.5c16195. 41385393

[21] Y. Jin , Y. Hu , M. Ortiz , S. Huang , Y. Ge , and W. Zhang , “Confined Growth of Ordered Organic Frameworks at an Interface,” Chemical Society Reviews 49, no. 14 (2020): 4637–4666, 10.1039/C9CS00879A. 32597423

[22] C. Liu , C.‐Y. Zhu , C. Zhang , H.‐C. Yang , and Z.‐K. Xu , “Thermodynamic and Kinetic Understanding for Managing the Controllability of Interfacial Polymerization,” Progress in Polymer Science 152 (2024): 101815, 10.1016/j.progpolymsci.2024.101815.

[23] Z. Wang , S. Liang , Y. Kang , et al., “Manipulating Interfacial Polymerization for Polymeric Nanofilms of Composite Separation Membranes,” Progress in Polymer Science 122 (2021): 101450, 10.1016/j.progpolymsci.2021.101450.

[24] X. Ma , T. Jia , X. An , and X. Zhan , “Multidimensional Regulation of Interfacial Polymerization for Structure‐Performance Synergistic Optimization of Polyamide Nanofiltration Membranes,” Journal of Environmental Chemical Engineering 13, no. 6 (2025): 119374, 10.1016/j.jece.2025.119374.

[25] Y. Pan , H. Liu , Z. Huang , et al., “Membranes Based on Covalent Organic Frameworks Through Green and Scalable Interfacial Polymerization Using Ionic Liquids for Antibiotic Desalination,” Angewandte Chemie International Edition 63, no. 4 (2024): 202316315, 10.1002/anie.202316315. 38030580

[26] T. Zhu , Y. Kong , B. Lyu , et al., “3D covalent Organic Framework Membrane With Fast and Selective Ion Transport,” Nature Communications 14, no. 1 (2023): 5926, 10.1038/s41467-023-41555-5. PMC10517170 37739946

[27] F. Yang , J. Guo , C. Han , et al., “Turing Covalent Organic Framework Membranes via Heterogeneous Nucleation Synthesis for Organic Solvent Nanofiltration,” Science Advances 10, no. 50 (2024): adr9260, 10.1126/sciadv.adr9260. PMC11633759 39661688

[28] J. Du , J. guan , A. A. AL‐Thuraya , et al., “Asymmetric Ionic Covalent Organic Framework Membranes With Tunable Turing Patterns Prepared via Ion Regulated Strategy,” Nature Communications 16, no. 1 (2025): 8664, 10.1038/s41467-025-63240-5. PMC12485139 41027864

[29] X. Li , X. Ji , X. Zhang , et al., “Construction of Functional Covalent Organic Framework Films by Modulator and Solvent Induced Polymerization,” Nature Communications 16, no. 1 (2025): 1223, 10.1038/s41467-024-55114-z. PMC11785801 39890837

[30] S. Liu , Z. Lin , C. Wang , and J. Guo , “Dual‐Sided Symmetric Crystalline Orientation of Covalent Organic Framework Membranes for Unidirectional Anhydrous Proton Conduction,” Science China Chemistry 65, no. 12 (2022): 2548–2557, 10.1007/s11426-022-1379-y.

[31] Y. Zou , Y. Zhang , J. Zhang , et al., “Cooperative Steric Modulation of Flexibility, Disorder, and Pore Size in Two‐Dimensional Covalent Organic Framework Membranes for Enhanced Selective Ion Sieving,” Journal of the American Chemical Society 147, no. 36 (2025): 32580–32590, 10.1021/jacs.5c07154. 40650601

[32] Y. Zhang , J. Guo , G. Han , et al., “Molecularly Soldered Covalent Organic Frameworks for Ultrafast Precision Sieving,” Science Advances 7, no. 13 (2021): abe8706, 10.1126/sciadv.abe8706. PMC7990329 33762342

[33] F. Liu , Z. Ma , Y. Deng , et al., “Tunable Covalent Organic Frameworks With Different Heterocyclic Nitrogen Locations for Efficient Cr(VI) Reduction, Escherichia coli Disinfection, and Paracetamol Degradation Under Visible‐Light Irradiation,” Environmental Science & Technology 55, no. 8 (2021): 5371–5381, 10.1021/acs.est.0c07857. 33739828

[34] Q. Liao , Q. Sun , H. Xu , et al., “Regulating Relative Nitrogen Locations of Diazine Functionalized Covalent Organic Frameworks for Overall H_2_O_2_ Photosynthesis,” Angewandte Chemie International Edition 62, no. 41 (2023): 202310556, 10.1002/anie.202310556. 37632257

[35] A. Taghipour , M. Dadashi Firouzjaei , C. Ammann , et al., “Unveiling the Impact of Monomer Reactivity on the Morphology and Functionality of Thin‐Film Composite Membranes,” Chemical Engineering Journal 480 (2024): 148028, 10.1016/j.cej.2023.148028.

[36] K. Huang , K. P. Reber , M. D. Toomey , J. A. Howarter , and A. D. Shah , “Reactivity of the Polyamide Membrane Monomer With Free Chlorine: Role of Bromide,” Environmental Science & Technology 55, no. 4 (2021): 2575–2584, 10.1021/acs.est.0c06122. 33497196

[37] Y.‐Q. Ding , Y.‐Z. Cui , and T.‐D. Li , “New Views on the Reaction of Primary Amine and Aldehyde from DFT Study,” The Journal of Physical Chemistry A 119, no. 18 (2015): 4252–4260, 10.1021/acs.jpca.5b02186. 25859816

[38] P. Flores‐Morales , S. Gutiérrez‐Oliva , E. Silva , and A. Toro‐Labbé , “The Reaction Electronic Flux: A New Descriptor of the Electronic Activity Taking Place During a Chemical Reaction. Application to the Characterization of the Mechanism of the Schiff's Base Formation in the Maillard Reaction,” Journal of Molecular Structure: THEOCHEM 943, no. 1‐3 (2020): 121–126, 10.1016/j.theochem.2009.11.013.

[39] X. Wang , B. Shi , H. Yang , et al., “Assembling Covalent Organic Framework Membranes With Superior Ion Exchange Capacity,” Nature Communications 13, no. 1 (2022): 1020, 10.1038/s41467-022-28643-8. PMC8866435 35197451

[40] Q. Liu , M. Liu , Z. Zhang , et al., “Covalent Organic Framework Membranes With Vertically Aligned Nanorods for Efficient Separation of Rare Metal Ions,” Nature Communications 15, no. 1 (2024): 9221, 10.1038/s41467-024-53625-3. PMC11511856 39455582

[41] X. Wu , P. Cheng , C. Cai , et al., “Physical and Chemical Dual Confinement Promotes Controllable Synthesis of Loose‐Structured Azine‐Linked Nanofilms for Fast Molecular Separation,” Nano Letters 24, no. 46 (2024): 14797–14805, 10.1021/acs.nanolett.4c04326. 39501765

[42] M. Sagisaka , T. Darmanin , F. Guittard , and J. Eastoe , “New Fluorine‐Free Low Surface Energy Surfactants and Surfaces,” Journal of Colloid and Interface Science 690 (2025): 137229, 10.1016/j.jcis.2025.03.018. 40112528

[43] F. Benyettou , A. Jrad , Z. Matouk , et al., “Tunable Wettability of a Dual‐Faced Covalent Organic Framework Membrane for Enhanced Water Filtration,” Journal of the American Chemical Society 146, no. 33 (2024): 23537–23554, 10.1021/jacs.4c07559. 39110940 PMC11345768

[44] Z.‐M. Han , Y. Hou , H.‐C. Liu , et al., “Fast and Massive Production of Aramid Nanofibers via Molecule Intercalation,” Journal of the American Chemical Society 147, no. 9 (2025): 7939–7949, 10.1021/jacs.4c18620. 39980472

[45] J. Zhu , M. Yang , A. Emre , et al., “Branched Aramid Nanofibers,” Angewandte Chemie International Edition 56, no. 39 (2017): 11744–11748, 10.1002/anie.201703766. 28722323

[46] A. Morelos‐Gomez , R. Cruz‐Silva , H. Muramatsu , et al., “Effective NaCl and Dye Rejection of Hybrid Graphene Oxide/Graphene Layered Membranes,” Nature Nanotechnology 12, no. 11 (2017): 1083–1088, 10.1038/nnano.2017.160. 28846102

[47] Q. Yang , Y. Su , C. Chi , et al., “Ultrathin Graphene‐Based Membrane With Precise Molecular Sieving and Ultrafast Solvent Permeation,” Nature Materials 16, no. 12 (2017): 1198–1202, 10.1038/nmat5025. 29170556

[48] M. Hu and B. Mi , “Enabling Graphene Oxide Nanosheets as Water Separation Membranes,” Environmental Science & Technology 47, no. 8 (2013): 3715–3723, 10.1021/es400571g. 23488812

[49] N. Wang , S. Ji , G. Zhang , J. Li , and L. Wang , “Self‐Assembly of Graphene Oxide and Polyelectrolyte Complex Nanohybrid Membranes for Nanofiltration and Pervaporation,” Chemical Engineering Journal 213 (2012): 318–329, 10.1016/j.cej.2012.09.080.

[50] Y. Han , Y. Jiang , and C. Gao , “High‐Flux Graphene Oxide Nanofiltration Membrane Intercalated by Carbon Nanotubes,” ACS Applied Materials & Interfaces 7, no. 15 (2015): 8147–8155, 10.1021/acsami.5b00986. 25837883

[51] Y. Han , Z. Xu , and C. Gao , “Ultrathin Graphene Nanofiltration Membrane for Water Purification,” Advanced Functional Materials 23, no. 29 (2013): 3693–3700, 10.1002/adfm.201202601.

[52] L. Ries , E. Petit , T. Michel , et al., “Enhanced Sieving from Exfoliated MoS_2_ Membranes via Covalent Functionalization,” Nature Materials 18, no. 10 (2019): 1112–1117, 10.1038/s41563-019-0464-7. 31451779

[53] H. Wang , J. Zhao , Y. Li , et al., “Aqueous Two‐Phase Interfacial Assembly of COF Membranes for Water Desalination,” Nano‐Micro Letters 14, no. 1 (2022): 216, 10.1007/s40820-022-00968-5. 36352333 PMC9646690

[54] L.‐P. Yue , F.‐X. Kong , Y. Wang , G.‐D. Sun , A.‐G. Zhou , and J.‐F. Chen , “Evolution of Morphology, Structure, and Performance of the COF Membranes With Monomer Concentrations: Enabling Effective Desalination and Kinetics Analysis,” Desalination 583 (2024): 117712, 10.1016/j.desal.2024.117712.

[55] R. Chen , S. Luo , Z. Song , et al., “Gradient‐Charged Cellulose Acetate Membranes Enabled by Ionic COF Nanosheets for Enhanced Nanofiltration‐Based Desalination,” Journal of Membrane Science 734 (2025): 124391, 10.1016/j.memsci.2025.124391.

[56] L.‐P. Yue , F.‐X. Kong , Y. Wang , G.‐D. Sun , and J.‐F. Chen , “PTSA‐Mediated Interfacial Catalytic Polymerization of Crystalline Dense Covalent Organic Framework Membranes for Enhanced Desalination,” Journal of Membrane Science 685 (2023): 121877, 10.1016/j.memsci.2023.121877.

[57] R. Wang , M. Wei , and Y. Wang , “Secondary Growth of Covalent Organic Frameworks (COFs) on Porous Substrates for Fast Desalination,” Journal of Membrane Science 604 (2020): 118090, 10.1016/j.memsci.2020.118090.

[58] F.‐X. Kong , L. Yue , Z. Yang , G. Sun , and J.‐F. Chen , “Cross‐Linked Covalent Organic Framework‐Based Membranes With Trimesoyl Chloride for Enhanced Desalination,” ACS Applied Materials & Interfaces 13, no. 18 (2021): 21379–21389, 10.1021/acsami.1c03628. 33914506

[59] J. Shen , J. Yuan , B. Shi , et al., “Homointerface Covalent Organic Framework Membranes for Efficient Desalination,” Journal of Materials Chemistry A 9, no. 40 (2021): 23178–23187, 10.1039/D1TA06439H.

[60] B. Khorshidi , T. Thundat , B. A. Fleck , and M. Sadrzadeh , “A Novel Approach Toward Fabrication of High Performance Thin Film Composite Polyamide Membranes,” Scientific Reports 6, no. 1 (2016): 22069, 10.1038/srep22069. 26924449 PMC4770410

[61] K. Guan , S. Fang , S. Zhou , et al., “Thin Film Composite Membrane With Improved Permeance for Reverse Osmosis and Organic Solvent Reverse Osmosis,” Journal of Membrane Science 688 (2023): 122104, 10.1016/j.memsci.2023.122104.

[62] S.‐L. Li , G. Chang , Y. Huang , et al., “2,2′‐Biphenol‐Based Ultrathin Microporous Nanofilms for Highly Efficient Molecular Sieving Separation,” Angewandte Chemie International Edition 61, no. 46 (2022): 202212816, 10.1002/anie.202212816. 36148532

[63] M. F. Jimenez‐Solomon , Q. Song , K. E. Jelfs , M. Munoz‐Ibanez , and A. G. Livingston , “Polymer Nanofilms With Enhanced Microporosity by Interfacial Polymerization,” Nature Materials 15, no. 7 (2016): 760–767, 10.1038/nmat4638. 27135857

