# Supplementary material for: Heteroatom‐Mediated Covalent Organic Framework Membranes With Nitrogen‐Enriched Nanochannels for Efficient Desalination
Source: Adv Sci (Weinh). 2026 Sep 13:e77400. Online ahead of print. doi: 10.1002/advs.77400 (PMC13572834; doi:10.1002/advs.77400)
Supplement: Supplementary file 1 [file ADVS-9999-e77400-s001.docx]

Supplementary Information

**Heteroatom-mediated covalent organic framework membranes with nitrogen-enriched nanochannels for efficient desalination**

*Guishan Hu**^1^, Muning Chen^1^, Yunqiu Zhou^1^, Yu Zhen^1^, Miaomiao Tian^2^, Yatao Zhang**^1^***, Jingwei Hou^3^, Junyong Zhu^1^*, Yong Wang^4^**

*^1^ School of Chemical Engineering, Zhengzhou University, Zhengzhou 450001, Henan, China*

*^2^ School of Ecology and Environment, Zhengzhou University, Zhengzhou, 450001, Henan, China.*

*^3^* *School of Chemical Engineering, The University of Queensland, StLucia, Queensland, Australia*

*^4^* *School of Energy and Environment, Southeast University, Nanjing, 210096, Jiangsu, China*

*Corresponding author. Email: zhangyatao@zzu.edu.cn; zhujunyong@zzu.edu.cn; yongwang@seu.edu.cn

Table of Contents

[1.	Supplementary Methods	5](#_Toc223203834)

[1.1 Membrane characterization.	5](#_Toc223203835)

[1.2 Monomer synthesis.	5](#_Toc223203836)

[1.2.1 Synthesis of PDHz precursors	5](#_Toc223203837)

[1.2.2 Synthesis of PAHz precursors	6](#_Toc223203838)

[1.3 Fabrication of COF free-standing nanofilm	7](#_Toc223203839)

[1.4 Fabrication of Kevlar hydrogel support membrane	7](#_Toc223203840)

[1.5 Desalination performance of COF membranes	8](#_Toc223203841)

[1.6 Mean pore size and pore size distribution	8](#_Toc223203842)

[1.7 Theoretical calculation method	9](#_Toc223203843)

[1.7.1 Computational assessment of monomer reactivity	9](#_Toc223203844)

[1.7.2 Molecular dynamics simulations of transport mechanisms	10](#_Toc223203845)

[2.	Morphology and Structure of Membranes	11](#_Toc223203846)

[Figure S1. The Optical photograph of the freestanding film at the free two-phase interface (Image of the film captured by a wire loop).	11](#_Toc223203847)

[Figure S2. FT-IR spectra of Tp-BDHz nanofilm, Tp-PDHz nanofilm, Tp-PAHz nanofilm, and raw materials.	11](#_Toc223203848)

[Figure S3. Solid-state ^13^C NMR spectrum of Tp-BDHz.	11](#_Toc223203849)

[Figure S4. SEM image of the transfer free-standing film (Tp-BDHz, Tp-PDHz, Tp-PAHz) on the surface of AAO substrate.	12](#_Toc223203850)

[Figure S5. TEM/ EDS mapping image and elemental distributions of the surface transfer free-standing film (Tp-BDHz).	13](#_Toc223203851)

[Figure S6. TEM/ EDS mapping image and elemental distributions of the surface transfer free-standing film (Tp-PDHz).	13](#_Toc223203852)

[Figure S7. TEM/ EDS mapping image and elemental distributions of the surface transfer free-standing film (Tp-PAHz).	14](#_Toc223203853)

[Figure S8. Reaction mechanism between Tp and hydrazide.	14](#_Toc223203854)

[Figure S9. Fukui functions of BDHz, PDHz, and PAHz.	15](#_Toc223203855)

[Figure S10. Comparison of XRD patterns of Tp-BDHz, Tp-PDHz, and Tp-PAHz self-supporting films and raw materials.	15](#_Toc223203856)

[Figure S11. Experimental, Pawley refined, and simulated PXRD profiles of the Tp-BDHz film with difference.	16](#_Toc223203857)

[Figure S12. Three possible stacking models for Tp-BDHz COF: (a) AA stacking model, (b) AB stacking model, (c) ABC stacking model.	16](#_Toc223203858)

[Figure S13. Three possible stacking models for Tp-PDHz COF: (a) AA stacking model, (b) AB stacking model, (c) ABC stacking model.	16](#_Toc223203859)

[Figure S14. Three possible stacking models for Tp-PAHz COF: (a) AA stacking model, (b) AB stacking model, (c) ABC stacking model.	17](#_Toc223203860)

[Figure S15. HRTEM image of Tp-BDHz.	17](#_Toc223203861)

[Figure S16. 2D GIWAXS data of Tp-BDHz.	17](#_Toc223203862)

[Figure S17. N_2_ adsorption-desorption isotherm and pore size distributions for the Tp-BDHz, Tp-PDHz and Tp-PAHz nanofilms	18](#_Toc223203863)

[Figure S18. Pore size distributions for the Tp-BDHz, Tp-PDHz and Tp-PAHz nanofilms	18](#_Toc223203864)

[Figure S19. The TGA curve of Tp-BDHz, Tp-PDHz, Tp-PAHz nanofilm.	19](#_Toc223203865)

[Figure S20. Tp-BDHz, Tp-PDHz, Tp-PAHz COF structures and composite membrane images.	19](#_Toc223203866)

[Figure S21. Comparison of FT-IR spectra of Tp-BDHz, Tp-PDHz, Tp-PAHz composite membranes and free-standing films.	20](#_Toc223203867)

[Figure S22. Adsorption curves of monomer BDHz, PDHz, and PAHz on Kevlar based membrane at different times	20](#_Toc223203868)

[Figure S23. The cross-sectional high-resolution TEM image of Tp-BDHz, Tp-PDHz and Tp-PAHz membranes.	21](#_Toc223203869)

[Figure S24. SEM image (a) and AFM image (b) of Kevlar substrate.	21](#_Toc223203870)

[Figure S25. Surface AFM image of Tp-BDHz, Tp-PDHz and Tp-PAHz membranes.	21](#_Toc223203871)

[Figure S26. SEM image of the Tp-BDHz membranes with different BDHz concentrations	22](#_Toc223203872)

[Figure S27. SEM image of the Tp-BDHz membranes with different Tp concentrations.	22](#_Toc223203873)

[Figure S28. SEM image of the Tp-PDHz membranes with different Tp concentrations.	23](#_Toc223203874)

[Figure S29. SEM image of the Tp-PDHz membranes with different PDHz concentrations.	23](#_Toc223203875)

[Figure S30. SEM image of the Tp-PAHz membranes with different Tp concentrations.	24](#_Toc223203876)

[Figure S31. SEM image of the Tp-PAHz membranes with different PAHz concentrations.	24](#_Toc223203877)

[Figure S32. SEM images of Tp-BDHz under different reaction times.	25](#_Toc223203878)

[Figure S33. SEM images of Tp-PDHz under different reaction times.	25](#_Toc223203879)

[Figure S34. SEM images of Tp-PAHz under different reaction times.	25](#_Toc223203880)

[Figure S35. XPS patterns of Kevlar hydrogel substrate, C1s spectrum, N1s spectrum, O1s spectrum.	26](#_Toc223203881)

[Figure S36. XPS patterns of Tp-BDHz membranes, C1s spectrum, N1s spectrum, O1s spectrum.	26](#_Toc223203882)

[Figure S37. XPS patterns of Tp-PDHz membranes, C1s spectrum, N1s spectrum, O1s spectrum.	26](#_Toc223203883)

[Figure S38. XPS patterns of Tp-PAHz membranes, C1s spectrum, N1s spectrum, O1s spectrum.	27](#_Toc223203884)

[Figure S39. Water permeance and salts rejection properties of Tp-PDHz membranes with different Tp/PDHz ratios.	27](#_Toc223203885)

[Figure S40. Water permeance and salts rejection properties of Tp-BDHz membranes with different BDHz concentration.	28](#_Toc223203886)

[Figure S41. Water permeance and salts rejection properties of Tp-BDHz membranes with different Tp concentration	28](#_Toc223203887)

[Figure S42. Water permeance and salts rejection properties of Tp-PAHz membranes with different PAHz concentration	29](#_Toc223203888)

[Figure S43. Water permeance and salts rejection properties of Tp-PAHz membranes with different Tp concentration	29](#_Toc223203889)

[Figure S44. Monitoring the dissipation of Tp during the membrane formation process of Tp-BDHz, Tp-PDHz, and Tp-PAHz using UV visible spectroscopy	30](#_Toc223203890)

[Figure S45. Curve fitting of dissipation rates for BDHz, PDHz, and PAHz	30](#_Toc223203891)

[Figure S46. Multi-synergistic mechanism in COF membrane fabrication	31](#_Toc223203892)

[Figure S47. Simulation setup for Tp-BDHz membranes, featuring a five-layer COF structure sandwiched between graphene pistons (orange) under a 15 bar pressure gradient, with water molecules shown in light blue.	31](#_Toc223203893)

[Figure S48. Simulated snapshots of Na⁺, Cl⁻, and H₂O molecules within the Tp-BDHz COF membranes at sequential time points	31](#_Toc223203894)

[Figure S49. Simulated ssnapshots of Na⁺, Cl⁻, and H₂O molecules within the Tp-PDHz COF membranes at sequential time points	32](#_Toc223203895)

[Figure S50. Cumulative number of H2O, Na+, and Cl- ions transferred across the COF membranes over time.	32](#_Toc223203897)

[Figure S51. Schematic diagram of interaction energies between Tp-BDHz and Na⁺/Cl⁻ ions	33](#_Toc223203898)

[Figure S52. Schematic diagram of interaction energies between Tp-PDHz and Na⁺/Cl⁻ ions	33](#_Toc223203899)

[Figure S53. The interfacial interactions between the COF membranes and H₂O/Na⁺/Cl⁻ species primarily involve electrostatic interactions and van der Waals forces.	34](#_Toc223203900)

[Figure S54. ^1^H NMR spectrum of PDHz	34](#_Toc223203901)

[Figure S55. HRMS spectrum of PDHz.	34](#_Toc223203902)

[Figure S56. ^1^H NMR spectrum of dimethyl pyrazine-2,5-dicarboxylate	35](#_Toc223203903)

[Figure S57. ^13^C NMR spectrum of dimethyl pyrazine-2,5-dicarboxylate	35](#_Toc223203904)

[Figure S58. ^1^H NMR spectrum of PAHz	35](#_Toc223203905)

[Figure S59. HRMS spectrum of PAHz.	36](#_Toc223203906)

[Table S1. Surface element compositions of different membranes were tested by XPS.	36](#_Toc223203907)

[Table S2 Summary of the membrane performance comparison for NaCl rejection.	37](#_Toc223203908)

[Supplementary references	38](#_Toc223203909)

1. Supplementary Methods

1.1 Membrane characterization.

The morphologies of the samples were characterized using field-emission scanning electron microscopy (SEM; Thermo Scientific Helios G4 CX), transmission electron microscopy (TEM; FEI Talos F200S), and atomic force microscopy (AFM; Bruker Dimension Fastscan). Powder X-ray diffraction (PXRD) patterns were recorded on a Panalytical instrument with a 2θ range of 2–50° and a step size of 0.02°. Fourier transform infrared (FT-IR) spectra were acquired on a Bruker VERTEX 70v spectrometer in the range of 4000–400 cm⁻¹ to identify organic functional groups. Solid-state MAS NMR spectroscopy (Bruker) was employed to analyze the chemical structure of the COF nanofilm. The surface elemental composition was determined by X-ray photoelectron spectroscopy (XPS; Thermo Scientific K-Alpha) using monochromatic Mg Kα radiation.

Thermal stability was evaluated by thermogravimetric analysis (TGA) on an STA 409 PC instrument under nitrogen atmosphere, with samples heated from 100 to 800 °C at a rate of 10 °C/min. Water contact angles (WCAs) were measured by depositing 5 μL of deionized water on the membrane surface using a contact angle goniometer. The specific surface area and pore size distribution of free-standing films were determined from N₂ sorption isotherms at 77 K using the Brunauer–Emmett–Teller (BET) method and nonlocal density functional theory (NLDFT), respectively. Zeta potentials at varying pH values were measured with a SurPASS^TM^ instrument (Anton Paar).

1.2 Monomer synthesis.

1.2.1 Synthesis of PDHz precursors

Scheme 1. Synthetic route for PDHz precursors.

The synthesis was performed under a nitrogen atmosphere. A 250 mL three-necked flask equipped with a reflux condenser was charged with dimethyl pyridine-2,5-dicarboxylate (5 g, 0.025 mol), followed by the addition of ethanol (50 mL) and hydrazine hydrate (80%, 6 mL). The reaction mixture was stirred and refluxed at 75 °C for 12 h. Upon completion, the mixture was cooled to room temperature, resulting in the precipitation of a yellow solid. The solid was collected by vacuum filtration and purified by washing thoroughly with ethanol (3 × 10 mL). The resulting product was dried under vacuum at 60 °C for 12 h to afford 4.6 g of PDHz as a light-yellow solid in 92% yield.

^1^H NMR (400MHz, DMSO-d6): δ (ppm) 10.06 (s, 1H, -N*H*-), 9,99 (s, 1H, -N*H*-), 8.97 (s, 1H, -Ph-*H*), 8.13 (d, J=8.0 Hz, 1H.-Ph-*H*), 8.11 (d, J=8.0 Hz, 1H.-Ph-*H*), 4,61 (s, 4H) (Figure S63).

High-resolution mass spectrometry (HRMS) (m/z): caculated for C_7_H_9_N_5_O_2_ [M+H]^+^: 196.1895, found: 196.1896 (Figure S64).

1.2.2 Synthesis of PAHz precursors

Scheme 2. Synthetic route for PAHz precursors.

Under a nitrogen atmosphere, a mixture of pyrazine-2,5-dicarboxylic acid dihydrate (5 g, 0.024 mol) in methanol (50 mL) and thionyl chloride (5.7 g, 0.048 mol) was stirred and refluxed at 75 °C for 12 h in a 250 mL three-necked flask. After cooling, the reaction was quenched by pouring it into 200 mL of ice-water. The product was extracted with dichloromethane (2 × 100 mL), and the combined organic layers were washed with deionized water to a neutral pH (6-7) and dried over anhydrous Na₂SO₄. The solution was concentrated under reduced pressure, and the product was recrystallized by adding hexane. The precipitated white solid was isolated by suction filtration, washed with hexane, and dried under vacuum at 60 °C for 12 h to yield 4.2 g (86%) of the intermediate.

^1^H NMR (400 MHz, DMSO-d6): δ (ppm) 9.31 (s, 2H, -Ph-*H*), 3,97 (s, 6H, -C*H_3_*) (Figure S65).

^13^C NMR (100 MHz, DMSO-d6): δ (ppm) 163.86, 145.52, 145.45, 53.43 (Figure S66).

Under a nitrogen atmosphere, a mixture of the intermediate (4 g, 0.02 mol) in ethanol (50 mL) and hydrazine hydrate (80%, 6 mL) was stirred and refluxed at 75 °C for 12 h in a 250 mL three-necked flask fitted with a reflux condenser. Following this, the mixture was cooled to room temperature, resulting in the precipitation of a tan solid. The crude product was isolated by vacuum filtration, washed thoroughly with ethanol, and dried under vacuum at 60 °C for 12 h, yielding 3.6 g (90%) of PAHz as a tan solid.

^1^H NMR (400 MHz, DMSO-d6): δ (ppm) 10.30 (s, 2H, -N*H*-), 9.11 (s, 2H, -Ph-*H*), 4.04(s, 4H, -N*H*_2_) (Figure S67).

High-resolution mass spectrometry (HRMS) (m/z): caculated for C_7_H_9_N_5_O_2_ [M+H]^+^: 197.0781, found: 197.0783 (Figure S68).

1.3 Fabrication of COF free-standing nanofilm

Free-standing COF nanofilms were synthesized under ambient conditions via interfacial polymerization. The organic phase was prepared by dissolving Tp (13.13 mg, 1.25 mM) in dichloromethane (DCM, 50 mL). The aqueous phase consisted of BDHz, PDHz, or PAHz (19.41, 19.52, or 19.62 mg, respectively, 2.0 mM) and HCl (125 μL) in water (50 mL). The aqueous solution was carefully layered over the organic phase in a Petri dish to establish a static liquid–liquid interface. The system was then sealed and allowed to react undisturbed for 24 hours.

1.4 Fabrication of Kevlar hydrogel support membrane

The Kevlar hydrogel support membrane was fabricated using a wet phase inversion method based on established protocols, involving three main steps: dissolution, casting, and phase inversion. Specifically, 1.2 g of Kevlar fibers were dissolved in a mixture of 60 mL DMSO, 1.2 mL deionized water, and 1.2 g KOH under continuous stirring at 25°C for 24 hours. The resulting dark red solution was then centrifuged at 8000 rpm for 30 minutes to remove air bubbles and any undissolved residues prior to membrane formation. A non-woven fabric substrate was secured on a clean glass plate, and the prepared solution was uniformly cast onto it with a controlled thickness of 250 μm. The cast film was immediately immersed in a coagulation bath of deionized water at 25°C for 10 minutes to induce phase separation. Finally, the formed Kevlar membrane was thoroughly rinsed in fresh deionized water for 6 hours to completely replace DMSO with water.

1.5 Desalination performance of COF membranes

At 25 °C, the water permeability and salt rejection of COF membranes, were tested via a cross-current device with a filtration area of 7.1 cm^2^. First the COF membranes were immobilized in scaffold modules was tested with DI sloution under 15 bar for 30 min. Then, the separation performance of the resulting membranes was tested at 15 bar. The water permeability (*P*, L m^−2^ h^−1^ bar^−1^) was determined using the following equation (1):

$P=\frac{V}{A\times\Delta P\times\Delta t}$	(1)

where *P* (L m^−2^ h^−1^ bar^−1^) is the permeability of the tested membrane, *V* (L) represents the permeate volume during operational time Δ*t* (h), *A* (m^2^) represents the desalination area, and Δ*P* (bar) is the applied pressure.

The conductivity meter (Thermo Scientific Orion Star A212) was used to determine the of salt ions concentration in feed and permeate fluids. The three salts (Na_2_SO_4_, MgCl_2_, and NaCl, the concentration is 2000 ppm) rejection of the prepared membranes were obtained by the following equation:

$R=(1-\frac{C_{P}}{C_{F}})\times100\%$	(2)

where *C_P_* and *C_F_* represent the conductivity of the permeate and feed fluids, respectively.

1.6 Mean pore size and pore size distribution

The molecular weight cutoff (MWCO) of the Pd^0^-TpPz membranes was characterized by conducting retention tests on polyethylene glycol (PEG) molecules with molecular weights of 106, 400, 600, 1000, and 1500 Da. A solution containing 1 g/L of PEG was tested at a pressure of 2 bar to determine the recovery rate. The MWCO of the COF membrane was defined as the molecular weight of PEG with a 90% rejection. The Stokes radius of the membrane pores was calculated using the average molecular weight of PEG according to Equation 3. ^1^

r = 16.73×10^-12^ MWCO^0.557^	(S3)

Based on the logarithmic normal model relating solute retention and solute Stokes radius, the solute retention quantity was linearly correlated with the diameter of polyethylene glycol. The membranes average effective pore size μp was defined as the Stokes radius of the solute at 50% rejection, and the geometric standard deviation σp was defined as the ratio between the diameter corresponding to 84.13% retention and the diameter at 50% rejection. Subsequently, the membrane pore size distribution was calculated using the probability density function based on the values of μp and σp according to Equation 4.

(S4)

1.7 Theoretical calculation method

1.7.1 Computational assessment of monomer reactivity

The electronic structure characteristics and chemical reactivity of monomer systems were investigated through first-principles density functional theory (DFT) calculations. All computations were performed within the Materials Studio software platform utilizing the DMol3 module. Structural optimization and electronic property evaluation employed the Perdew-Burke-Ernzerhof (PBE) functional under the generalized gradient approximation (GGA) framework, with a double numerical plus polarization (DNP) basis set implemented to ensure computational accuracy. Analysis of electrostatic potential distributions revealed distinct nucleophilic and electrophilic regions on molecular surfaces. Charge density distributions and frontier molecular orbital energy levels were calculated to elucidate electron delocalization behavior and identify potential reactive sites. Furthermore, condensed Fukui functions based on Hirshfeld population analysis provided quantitative assessment of nucleophilic and electrophilic reactivity at individual atomic positions from a kinetic perspective. These computational approaches collectively establish a theoretical framework for understanding selective behavior in interfacial polymerization processes.

1.7.2 Molecular dynamics simulations of transport mechanisms

Molecular dynamics simulations were conducted to investigate the transport mechanisms of ions and water molecules through covalent organic framework membranes. Both COF structures were modeled using the GAFF2 force field ^2^, with atomic charges derived from CP2K ^3^ calculations employing the REPEAT method. The OPC3 water model was implemented for water molecules, while ion parameters were adopted from literature values previously optimized for OPC3 aqueous environments ^4,5^. The simulation system incorporated graphene pistons on both membrane sides to establish pressure-driven transport conditions. The simulation system, constructed using GROMACS ^6^, consisted of 13,311 water molecules and 87 Na⁺/Cl⁻ ion pairs in the feed chamber, with 15,638 water molecules in the permeate chamber. All simulations were performed using GROMACS 2023.3 with a 2 fs integration time step. Following energy minimization, production simulations were carried out for 400 ns under the NVT ensemble. Temperature was maintained at 298.15 K using the V-rescale thermostat, with van der Waals interactions truncated at 12 Å and electrostatic interactions treated using the particle mesh Ewald method. Trajectories were saved every 10 ps and visualized using VMD software ^7^. Mean squared displacement (MSD) analysis was employed to characterize the diffusion behavior of Na⁺, Cl⁻, and H₂O within different COF structures. The potential of mean force (PMF) for transmembrane transport was calculated using umbrella sampling, with the reaction coordinate defined along the membrane normal direction from 1.0 to 5.0 nm. A harmonic restraint with a force constant of 2000 kJ·mol⁻¹·nm⁻² was applied, and the reaction path was divided into 40 windows, each simulated for 4 ns, resulting in a cumulative sampling time of 160 ns. The weighted histogram analysis method was subsequently used to compute the free energy profiles.

2. Morphology and Structure of Membranes

Figure S1. The Optical photograph of the freestanding film at the free two-phase interface (Image of the film captured by a wire loop).

Figure S2. FT-IR spectra of Tp-BDHz nanofilm, Tp-PDHz nanofilm, Tp-PAHz nanofilm, and raw materials.

Figure S3. Solid-state ^13^C NMR spectrum of Tp-BDHz.

Figure S4. SEM image of the transfer free-standing film (Tp-BDHz, Tp-PDHz, Tp-PAHz) on the surface of AAO substrate.

.

Figure S5. TEM/ EDS mapping image and elemental distributions of the surface transfer free-standing film (Tp-BDHz).

Figure S6. TEM/ EDS mapping image and elemental distributions of the surface transfer free-standing film (Tp-PDHz).

Figure S7. TEM/ EDS mapping image and elemental distributions of the surface transfer free-standing film (Tp-PAHz).

Figure S8. Reaction mechanism between Tp and hydrazide.

Figure S9. Fukui functions of BDHz, PDHz, and PAHz.

Figure S10. Comparison of XRD patterns of Tp-BDHz, Tp-PDHz, and Tp-PAHz self-supporting films and raw materials.

Figure S11. Experimental, Pawley refined, and simulated PXRD profiles of the Tp-BDHz film with difference.

Figure S12. Three possible stacking models for Tp-BDHz COF: (a) AA stacking model, (b) AB stacking model, (c) ABC stacking model.

Figure S13. Three possible stacking models for Tp-PDHz COF: (a) AA stacking model, (b) AB stacking model, (c) ABC stacking model.

Figure S14. Three possible stacking models for Tp-PAHz COF: (a) AA stacking model, (b) AB stacking model, (c) ABC stacking model.

.

Figure S15. Pawley refinement of Tp-PDHz COF PXRD patterns using AA (top), AB (middle), and ABC (bottom) stacking models.

Figure S16. Pawley refinement of Tp-PAHz COF PXRD patterns using AA (top), AB (middle), and ABC (bottom) stacking models.

Figure S17. Pawley refinement of Tp-BDHz COF PXRD patterns using AA (top), AB (middle), and ABC (bottom) stacking models.

Figure S18. HRTEM image of Tp-BDHz

Figure S19. High-resolution TEM images of Tp-BDHz, Tp-PDHz and Tp-PAHz COF membranes with Fourier filtering analysis.

Figure S20. 2D GIWAXS data of Tp-BDHz.

Figure S21. N_2_ adsorption-desorption isotherm and pore size distributions for the Tp-BDHz, Tp-PDHz and Tp-PAHz nanofilms

Figure S22. Pore size distributions for the Tp-BDHz, Tp-PDHz and Tp-PAHz nanofilms

Figure S23. The TGA curve of Tp-BDHz, Tp-PDHz, Tp-PAHz nanofilm.

Figure S24. Tp-BDHz, Tp-PDHz, Tp-PAHz COF structures and composite membrane images.

Figure S25. Comparison of FT-IR spectra of Tp-BDHz, Tp-PDHz, Tp-PAHz composite membranes and free-standing films.

Figure S26. Adsorption curves of monomer BDHz, PDHz, and PAHz on Kevlar based membrane at different times

Figure S27. The cross-sectional high-resolution TEM image of Tp-BDHz, Tp-PDHz and Tp-PAHz membranes.

Figure S28. SEM image (a) and AFM image (b) of Kevlar substrate.

Figure S29. Surface AFM image of Tp-BDHz, Tp-PDHz and Tp-PAHz membranes.

Figure S30. SEM image of the Tp-BDHz membranes with different BDHz concentrations

Figure S31. SEM image of the Tp-BDHz membranes with different Tp concentrations.

Figure S32. SEM image of the Tp-PDHz membranes with different Tp concentrations.

Figure S33. SEM image of the Tp-PDHz membranes with different PDHz concentrations.

Figure S34. SEM image of the Tp-PAHz membranes with different Tp concentrations.

Figure S35. SEM image of the Tp-PAHz membranes with different PAHz concentrations.

Figure S36. SEM images of Tp-BDHz under different reaction times.

Figure S37. SEM images of Tp-PDHz under different reaction times.

Figure S38. SEM images of Tp-PAHz under different reaction times.

Figure S39. XPS patterns of Kevlar hydrogel substrate, C1s spectrum, N1s spectrum, O1s spectrum.

Figure S40. XPS patterns of Tp-BDHz membranes, C1s spectrum, N1s spectrum, O1s spectrum.

Figure S41. XPS patterns of Tp-PDHz membranes, C1s spectrum, N1s spectrum, O1s spectrum.

Figure S42. XPS patterns of Tp-PAHz membranes, C1s spectrum, N1s spectrum, O1s spectrum.

z

Figure S43. Water permeance and salts rejection properties of Tp-PDHz membranes with different Tp/PDHz ratios.

Figure S44. Water permeance and salts rejection properties of Tp-BDHz membranes with different Tp concentration.

Figure S45. Water permeance and salts rejection properties of Tp-BDHz membranes with different Tp concentration

Figure S46. Water permeance and salts rejection properties of Tp-PAHz membranes with different PAHz concentration

Figure S47. Water permeance and salts rejection properties of Tp-PAHz membranes with different Tp concentration

Figure S48. Comparison of rejection rates of the Tp-PDHz membrane toward different salt solutions (feed concentration: 2000 ppm, operating pressure: 15 bar, temperature: 25 ℃)

.

Figure S49. Digital photographs of Tp-PDHz membranes prepared on different porous substrates: (a) Kevlar, (b) PES, (c) PVDF, and (d) PAN.

Figure S50. Water permeance and NaCl rejection of Tp-PDHz membranes fabricated on different porous supports (Kevlar, PES, PVDF, and PAN) under identical interfacial polymerization conditions (feed: 2000 ppm NaCl, pressure: 15 bar, temperature: 25 °C).

Figure S51. Water flux of the Tp-PDHz membrane as a function of applied pressure (feed: 2000 ppm NaCl, cross-flow mode, 25 °C).

Figure S52. Intrinsic water permeability coefficient (A) and true salt rejection of the Tp-PDHz membrane as a function of applied pressure.

Figure S53. Monitoring the dissipation of Tp during the membrane formation process of Tp-BDHz, Tp-PDHz, and Tp-PAHz using UV visible spectroscopy

Figure S54. Curve fitting of dissipation rates for BDHz, PDHz, and PAHz

Figure S55. Multi-synergistic mechanism in COF membrane fabrication

Figure S56. Simulation setup for Tp-BDHz membranes, featuring a five-layer COF structure sandwiched between graphene pistons (orange) under a 15 bar pressure gradient, with water molecules shown in light blue.

Figure S57. Simulated snapshots of Na⁺, Cl⁻, and H₂O molecules within the Tp-BDHz COF membranes at sequential time points

Figure S58. Simulated ssnapshots of Na⁺, Cl⁻, and H₂O molecules within the Tp-PDHz COF membranes at sequential time points

Figure S59. Cumulative number of H2O, Na+, and Cl- ions transferred across the COF membranes over time.

Figure S60. Schematic diagram of interaction energies between Tp-BDHz and Na⁺/Cl⁻ ions

Figure S61. Schematic diagram of interaction energies between Tp-PDHz and Na⁺/Cl⁻ ions

Figure S62. The interfacial interactions between the COF membranes and H₂O/Na⁺/Cl⁻ species primarily involve electrostatic interactions (Coul) and van der Waals forces (LJ).

Figure S63. ^1^H NMR spectrum of PDHz

Figure S64. HRMS spectrum of PDHz.

Figure S65. ^1^H NMR spectrum of dimethyl pyrazine-2,5-dicarboxylate

Figure S66. ^13^C NMR spectrum of dimethyl pyrazine-2,5-dicarboxylate

Figure S67. ^1^H NMR spectrum of PAHz

Figure S68. HRMS spectrum of PAHz.

Table S1. Surface element compositions of different membranes were tested by XPS.

| Membranes | Surface element composition (%) | | |  |
| --- | --- | --- | --- | --- |
|  | C | N | O | |
| Kevlar substrate | 74.82 | 10.11 | 15.07 | |
| Tp-BDHz Membrane | 69.43 | 8.6 | 21.96 | |
| Tp-PDHz membrane | 67.92 | 7.36 | 24.72 | |
| Tp-PAHz membrane | 64.84 | 12.99 | 22.18 | |

Table S2 Summary of the membrane performance comparison for NaCl rejection.

| Membranes | Water permeance  (LMH bar^-1^) | R_NaCl_ (%) | | Ref | |  |
| --- | --- | --- | --- | --- | --- | --- |
| Graphene/GO | 0.34 | | 85 | | (8) | |
| Laminated GO | 4 | | 25 | | (9) | |
| GO/TMC | 50 | | 19 | | (10) | |
| GO/PECs | 0.8 | | 43 | | (11) | |
| Graphene/CNT | 11.3 | | 51 | | (12) | |
| Graphene | 3.26 | | 42 | | (13) | |
| MoS_2_ | 1.6 | | 87.6 | | (14) | |
| COF-DhTG_Cl_ | 3.9 | | 93 | | (15) | |
| TpPa | 11.2 | | 26.2 | | (16) | |
| TpPa-SO_3_H | 0.43 | | 75 | | (17) | |
| TpPa (4) | 18.65 | | 38.3 | | (18) | |
| TpHz (5) | 40.5 | | 6.7 | | (19) | |
| PaTP_0.05%_−TMC_0.2%/30_ | 0.81 | | 93.3 | | (20) | |
| TpPa–SO_3_H/TpPa–SO_3_H/MPAN | 13.1 | | 45.3 | | (21) | |
| PES-(TMC-MPD) | 2.4 | | 93.2 | | (22) | |
| PK/PSf-(TMC-MPD) | 0.58 | | 98 | | (23) | |
| This Work (BDHz) | 1.3 | | 95.0 | | This Work | |
| This Work (PDHz) | 1,2 | | 97.5 | | This Work | |
| This Work (PAHz) | 1.4 | | 93.1 | | This Work | |

Supplementary references

(1) Hu, G.; Zhou, B.; Zhen, Y.; Zhu, J.; Hou, J.; Zhang, Y.; Wang, Y.; Van der Bruggend, B. Covalent organic framework catalytic membranes for durable multitasking water purification. *Chemical Science* **2025**, *16* (39), 18141-18151, 10.1039/D5SC04204F. DOI: 10.1039/D5SC04204F.

(2) Wang, J.; Wolf, R. M.; Caldwell, J. W.; Kollman, P. A.; Case, D. A. Development and testing of a general amber force field. *J. Comput. Chem.* **2004**, *25* (9), 1157-1174. DOI: https://doi.org/10.1002/jcc.20035 (acccessed 2025/11/28).

(3) Kühne, T. D.; Iannuzzi, M.; Del Ben, M.; Rybkin, V. V.; Seewald, P.; Stein, F.; Laino, T.; Khaliullin, R. Z.; Schütt, O.; Schiffmann, F.; et al. CP2K: An electronic structure and molecular dynamics software package - Quickstep: Efficient and accurate electronic structure calculations. *The Journal of Chemical Physics* **2020**, *152* (19), 194103. DOI: 10.1063/5.0007045 (acccessed 11/29/2025).

(4) Izadi, S.; Onufriev, A. V. Accuracy limit of rigid 3-point water models. *The Journal of Chemical Physics* **2016**, *145* (7), 074501. DOI: 10.1063/1.4960175 (acccessed 11/29/2025).

(5) Sengupta, A.; Li, Z.; Song, L. F.; Li, P.; Merz, K. M., Jr. Parameterization of Monovalent Ions for the OPC3, OPC, TIP3P-FB, and TIP4P-FB Water Models. *Journal of Chemical Information and Modeling* **2021**, *61* (2), 869-880. DOI: 10.1021/acs.jcim.0c01390.

(6) Abraham, M. J.; Murtola, T.; Schulz, R.; Páll, S.; Smith, J. C.; Hess, B.; Lindahl, E. GROMACS: High performance molecular simulations through multi-level parallelism from laptops to supercomputers. *SoftwareX* **2015**, *1-2*, 19-25. DOI: https://doi.org/10.1016/j.softx.2015.06.001.

(7) Humphrey, W.; Dalke, A.; Schulten, K. VMD: Visual molecular dynamics. *Journal of Molecular Graphics* **1996**, *14* (1), 33-38. DOI: <https://doi.org/10.1016/0263-7855(96)00018-5>.

(8) Morelos-Gomez, A.; Cruz-Silva, R.; Muramatsu, H.; Ortiz-Medina, J.; Araki, T.; Fukuyo, T.; Tejima, S.; Takeuchi, K.; Hayashi, T.; Terrones, M.; et al. Effective NaCl and dye rejection of hybrid graphene oxide/graphene layered membranes. *Nature Nanotechnology* **2017**, *12* (11), 1083-1088. DOI: 10.1038/nnano.2017.160.

(9) Yang, Q.; Su, Y.; Chi, C.; Cherian, C. T.; Huang, K.; Kravets, V. G.; Wang, F. C.; Zhang, J. C.; Pratt, A.; Grigorenko, A. N.; et al. Ultrathin graphene-based membrane with precise molecular sieving and ultrafast solvent permeation. *Nature Materials* **2017**, *16* (12), 1198-1202. DOI: 10.1038/nmat5025.

(10) Hu, M.; Mi, B. Enabling Graphene Oxide Nanosheets as Water Separation Membranes. *Environmental Science & Technology* **2013**, *47* (8), 3715-3723. DOI: 10.1021/es400571g.

(11) Wang, N.; Ji, S.; Zhang, G.; Li, J.; Wang, L. Self-assembly of graphene oxide and polyelectrolyte complex nanohybrid membranes for nanofiltration and pervaporation. *Chem. Eng. J.* **2012**, *213*, 318-329. DOI: https://doi.org/10.1016/j.cej.2012.09.080.

(12) Han, Y.; Jiang, Y.; Gao, C. High-Flux Graphene Oxide Nanofiltration Membrane Intercalated by Carbon Nanotubes. *ACS Applied Materials & Interfaces* **2015**, *7* (15), 8147-8155. DOI: 10.1021/acsami.5b00986.

(13) Han, Y.; Xu, Z.; Gao, C. Ultrathin Graphene Nanofiltration Membrane for Water Purification. *Adv. Funct. Mater.* **2013**, *23* (29), 3693-3700. DOI: https://doi.org/10.1002/adfm.201202601 (acccessed 2025/11/29).

(14) Ries, L.; Petit, E.; Michel, T.; Diogo, C. C.; Gervais, C.; Salameh, C.; Bechelany, M.; Balme, S.; Miele, P.; Onofrio, N.; et al. Enhanced sieving from exfoliated MoS2 membranes via covalent functionalization. *Nature Materials* **2019**, *18* (10), 1112-1117. DOI: 10.1038/s41563-019-0464-7.

(15) Wang, H.; Zhao, J.; Li, Y.; Cao, Y.; Zhu, Z.; Wang, M.; Zhang, R.; Pan, F.; Jiang, Z. Aqueous Two-Phase Interfacial Assembly of COF Membranes for Water Desalination. *Nano-Micro Letters* **2022**, *14* (1), 216. DOI: 10.1007/s40820-022-00968-5.

(16) Yue, L.-p.; Kong, F.-x.; Wang, Y.; Sun, G.-d.; Zhou, A.-g.; Chen, J.-f. Evolution of morphology, structure, and performance of the COF membranes with monomer concentrations: Enabling effective desalination and kinetics analysis. *Desalination* **2024**, *583*, 117712. DOI: https://doi.org/10.1016/j.desal.2024.117712.

(17) Chen, R.; Luo, S.; Song, Z.; Tian, Y.; Shi, D.; Lu, B.; Liu, H.; Tang, L.; Lin, G.; Shi, J.; et al. Gradient-charged cellulose acetate membranes enabled by ionic COF nanosheets for enhanced nanofiltration-based desalination. *J. Membr. Sci.* **2025**, *734*, 124391. DOI: https://doi.org/10.1016/j.memsci.2025.124391.

(18) Yue, L.-p.; Kong, F.-x.; Wang, Y.; Sun, G.-d.; Chen, J.-f. PTSA-mediated interfacial catalytic polymerization of crystalline dense covalent organic framework membranes for enhanced desalination. *J. Membr. Sci.* **2023**, *685*, 121877. DOI: https://doi.org/10.1016/j.memsci.2023.121877.

(19) Wang, R.; Wei, M.; Wang, Y. Secondary growth of covalent organic frameworks (COFs) on porous substrates for fast desalination. *J. Membr. Sci.* **2020**, *604*, 118090. DOI: https://doi.org/10.1016/j.memsci.2020.118090.

(20) Kong, F.-x.; Yue, L.; Yang, Z.; Sun, G.; Chen, J.-f. Cross-Linked Covalent Organic Framework-Based Membranes with Trimesoyl Chloride for Enhanced Desalination. *ACS Applied Materials & Interfaces* **2021**, *13* (18), 21379-21389. DOI: 10.1021/acsami.1c03628.

(21) Shen, J.; Yuan, J.; Shi, B.; You, X.; Ding, R.; Zhang, T.; Zhang, Y.; Deng, Y.; Guan, J.; Long, M.; et al. Homointerface covalent organic framework membranes for efficient desalination. *Journal of Materials Chemistry A* **2021**, *9* (40), 23178-23187, 10.1039/D1TA06439H. DOI: 10.1039/D1TA06439H.

(22) Khorshidi, B.; Thundat, T.; Fleck, B. A.; Sadrzadeh, M. A Novel Approach Toward Fabrication of High Performance Thin Film Composite Polyamide Membranes. *Scientific Reports* **2016**, *6* (1), 22069. DOI: 10.1038/srep22069.

(23) Guan, K.; Fang, S.; Zhou, S.; Fu, W.; Li, Z.; Gonzales, R. R.; Xu, P.; Mai, Z.; Hu, M.; Zhang, P.; et al. Thin film composite membrane with improved permeance for reverse osmosis and organic solvent reverse osmosis. *J. Membr. Sci.* **2023**, *688*, 122104. DOI: https://doi.org/10.1016/j.memsci.2023.122104.
